# *Labiobaetis* Novikova & Kluge in Borneo (Ephemeroptera, Baetidae)

**DOI:** 10.3897/zookeys.914.47067

**Published:** 2020-02-20

**Authors:** Thomas Kaltenbach, Jean-Luc Gattolliat

**Affiliations:** 1 Museum of Zoology, Palais de Rumine, Place Riponne 6, CH-1005 Lausanne, Switzerland Museum of Zoology Lausanne Switzerland; 2 University of Lausanne (UNIL), Department of Ecology and Evolution, CH-1015 Lausanne, Switzerland University of Lausanne Lausanne Switzerland

**Keywords:** Brunei, COI, imagos, Indonesia, Malaysia, new species, Southeast Asia

## Abstract

Material collected between 2000 and 2014 on the island Borneo, including the Indonesian province of Kalimantan, the Malaysian province of Sabah and Brunei Darussalam, substantially increased our knowledge of *Labiobaetis* on this island. The total number of *Labiobaetis* species in Borneo increased to five, as only one species, *L. borneoensis* (Müller-Liebenau, 1984), was previously reported. Three new species were identified by morphology and partly by using genetic distance (COI, Kimura 2-parameter). They are described and illustrated based on their larvae (*Labiobaetis bakerae* sp. nov., *L. penan* sp. nov. and *L. dayakorum* sp. nov.); in one case, the imago is described as well. New reports of *L. borneoensis* are presented and the imago of this species is described for the first time. *Labiobaetis moriharai* (Müller-Liebenau, 1984), originally described from mainland Malaysia (Province Selangor), is reported from Borneo for the first time. The interspecific K2P distances in Borneo are between 19% and 25%, the intraspecific distances are usually between 0% and 1%. The total number of *Labiobaetis* species worldwide is augmented to 126.

## Introduction

The family Baetidae has the highest species diversity among mayflies, comprising 1,070 species in 110 genera (Sartori and Brittain 2015, Jacobus et al. 2019), which is approx. one quarter of all mayfly species worldwide (Gattolliat and Nieto 2009, Jacobus et al. 2019). They have a cosmopolitan distribution except Antarctica and New Zealand. Investigations of the molecular phylogeny of the Order Ephemeroptera revealed the relatively primitive status of the family (Ogden and Whiting 2005, Ogden et al. 2009).

The genus *Labiobaetis* Novikova and Kluge (Novikova and Kluge 1987) is one of the richest genera of Baetidae with previously 123 described species (Barber-James et al. 2013, Webb 2013, Shi and Tong 2014, Kubendran et al. 2014, 2015, Gattolliat et al. 2018, Kaltenbach and Gattolliat 2018, 2019). The distribution of *Labiobaetis* is nearly worldwide, with the exception of the Neotropical realm, New Zealand and New Caledonia. The status and validity of the genus has often been a subject of controversy for a long time, but nowadays *Labiobaetis* is widely accepted as a valid genus (Gattolliat 2001, Fujitani et al. 2003, Fujitani 2008, McCafferty et al. 2010, Gattolliat and Staniczek 2011, Kluge and Novikova 2011, 2014, 2016, Kluge 2012, Webb 2013, Kubendran et al. 2014, 2015, Shi and Tong 2014). The history and concept of the genus *Labiobaetis* were recently summarized in detail (Shi and Tong 2014, Kaltenbach and Gattolliat 2018). All Oriental species previously transferred to *Pseudocloeon* (Lugo-Ortiz et al. 1999) were formerly reassigned to *Labiobaetis* by Shi and Tong (2014). Molecular reconstructions indicated that the concept of *Labiobaetis* is probably at least diphyletic (Monaghan et al. 2005, Gattolliat et al. 2008).

Borneo is the third largest island after Greenland and New Guinea. It forms part of the Sundaland Biodiversity Hotspot comprising Borneo, Sumatra, Java, and the Malay Peninsula and lies at the equator, reaching from 7°N to approx. 4°S, directly West of Wallace’s Line (Quek 2010). Borneo belongs to three different countries, the largest part by far in the South and West belongs to Indonesia (Province Kalimantan), another substantial part belongs to Malaysia (Provinces Sabah and Sarawak) and a very small part in the North is Brunei Darussalam. Geomorphically, Borneo is characterised by a central mountain massif with its highest peak, Mt. Kinabalu (4,095 m), in the north, and otherwise, more than half of the island lies below 150 m (Quek 2010). Borneo’s biota is very rich, influenced by a dynamic and highly complex geophysical history of the Sunda Shelf, including changing climates, fluctuating sea levels, volcanism and orogenic activity with subsequent erosion (Quek 2010). During an 85 km^2^ survey of the mayfly fauna of a lowland tropical forest in Borneo more than 40 mayfly genera were collected and at least ten new genera and many new species were discovered (Derleth 2003, Sartori et al. 2003).

So far, the diversity of *Labiobaetis* in Borneo was poorly known, as only one species was reported (*L. borneoensis* by Müller-Liebenau 1984b). Here, we increase the total number of *Labiobaetis* species in Borneo to five, based on material collected between 2000 and 2014 in ca. 20 different localities, which belong to four different areas in Borneo (Fig. 15). We describe three new species of *Labiobaetis*, one at larval and imaginal stage, the other two based on larvae only. Additionally, we have new reports of *L. borneoensis* (Müller-Liebenau) and we describe the imago of this species for the first time. We also report another species for the first time from Borneo (*L. moriharai*), so far known from mainland Malaysia (Prov. Selangor, Müller-Liebenau 1984a) and Vietnam (Soldán 1991).

## Materials and methods

The specimens from Indonesia (Kalimantan) were collected by Pascale Derleth-Sartori and colleagues (Museum of Zoology Lausanne, MZL; Derleth 2003). Further material was collected by Hendrik Freitag and his team (Ateneo de Manila University), and by Kate Baker (University of Exeter, UK) during ecological studies in Brunei Darussalam in collaboration with Universiti Brunei Darussalam (Baker et al. 2016a, b, 2017a, b).

The specimens were preserved in 70%–96% ethanol. The dissection of larvae was done in Cellosolve (2-Ethoxyethanol) with subsequent mounting on slides with Euparal liquid, using an Olympus SZX7 stereomicroscope.

The DNA of part of the specimens was extracted using non-destructive methods allowing subsequent morphological analysis (see Vuataz et al. 2011 for details). We amplified a 658 bp fragment of the mitochondrial gene cytochrome oxidase subunit 1 (COI) using the primers LCO 1490 (GGTCAACAAATCATAAAGATATTGG) and HCO 2198 (TAAACTTCAGGGTGACCAAAAAATCA) (Folmer et al. 1994). The polymerase chain reaction was conducted with an initial denaturation temperature of 98 °C for 30 sec followed by a total of 37 cycles with denaturation temperature of 98 °C for 10 sec, an annealing temperature of 50 °C for 30 sec and an extension at 72 °C for 30 sec, final extension at 72 °C for 2 min. Sequencing was done with Sanger’s method (Sanger et al. 1977). The genetic variability between specimens was estimated using Kimura 2-parameter distances (K2P, Kimura 1980), calculated with the program MEGA 7 (Kumar et al. 2016, http://www.megasoftware.net). The GenBank accession numbers are given in Table 1, nomenclature of gene sequences follows Chakrabarty et al. (2013).

**Table 1.** Sequenced specimens.

Species	Locality		Specimens catalog #	GenBank # (COI)	GenSeq Nomenclature
*L. bakerae* sp. nov.	Brunei	larva	GBIFCH 00592299	MN482248	genseq-2 COI
		larva	GBIFCH 00658084	MN482250	genseq-2 COI
		larva	GBIFCH 00592282	MN482249	genseq-2 COI
*L. penan* sp. nov.	Malaysia: Sabah	larva	GBIFCH 00654918	MN482251	genseq-2 COI
		larva	GBIFCH 00672299	MN482252	genseq-1 COI
		imago	GBIFCH 00672296	MN482253	genseq-2 COI
*L. borneoensis* (Müller-Liebenau)	Malaysia: Sabah	larva	GBIFCH 00658081	MN482254	genseq-4 COI
		imago	GBIFCH 00672289	MN482255	genseq-4 COI
*L. moriharai* (Müller-Liebenau)	Brunei	larva	GBIFCH 00658106	MN482256	genseq-4 COI

Drawings were made using an Olympus BX43 microscope. Photographs of larvae were taken using a Canon EOS 6D camera and the Visionary Digital Passport imaging system (http://www.duninc.com) and processed with the programs Adobe Photoshop Lightroom (http://www.adobe.com) and Helicon Focus version 5.3 (http://www.heliconsoft.com). Photographs were subsequently enhanced with Adobe Photoshop Elements 13.

The distribution maps were generated with the program SimpleMappr (https://simplemappr.netShorthouse 2010), the program GEOLocate (http://www.museum.tulane.edu/geolocate/web/WebGeoref.aspx) and Google Earth (http://www.google.com/earth/download/ge/) were used to attribute approximate GPS coordinates to sample locations of Müller-Liebenau (1984a, b) and Soldán (1991).

The taxonomic descriptions were generated with a DELTA (Dallwitz 1980, Dallwitz et al. 1999, Coleman et al. 2010) database containing the morphological states of characters of the *Labiobaetis* species of Borneo.

The terminology follows Hubbard (1995), Morihara and McCafferty (1979), and Kluge (2004). The postero-lateral extension of the paraproct is termed cercotractor following Kluge (2004).

## Results

### New species descriptions

Abbreviations:

**MZL**Museum of Zoology Lausanne (Switzerland)

**PNM**Museum of Natural History of the Philippine National Museum, Manila (Philippines)

#### *Labiobaetis sumigarensis* group of species (Müller-Liebenau 1982, Müller-Liebenau and Hubbard 1985, Kaltenbach and Gattolliat 2019)

Following combination of characters: A) dorsal surface of labrum with submarginal arc of clavate, apically smooth setae; B) labial palp segment II with large, lobed or thumb-like distomedial protuberance, outer margin of protuberance predominantly concave (*L. sumigarensis* with hook-like modification of the protuberance); C) left mandible without setae at apex of mola, with minute denticles between prostheca and mola; D) six pairs of gills; E) hindwing pads absent; F) distolateral process at scape poorly developed or absent; G) colour of larvae dorsally uniform brown.

##### Labiobaetis bakerae sp. nov.

Taxon classificationAnimaliaEphemeropteraBaetidae

0FFBEDDE-CE8E-5002-BE42-573A9CA558E8

http://zoobank.org/8394FCC0-7343-44F8-B6BF-D06FC34B30C0

**Figure 1.**
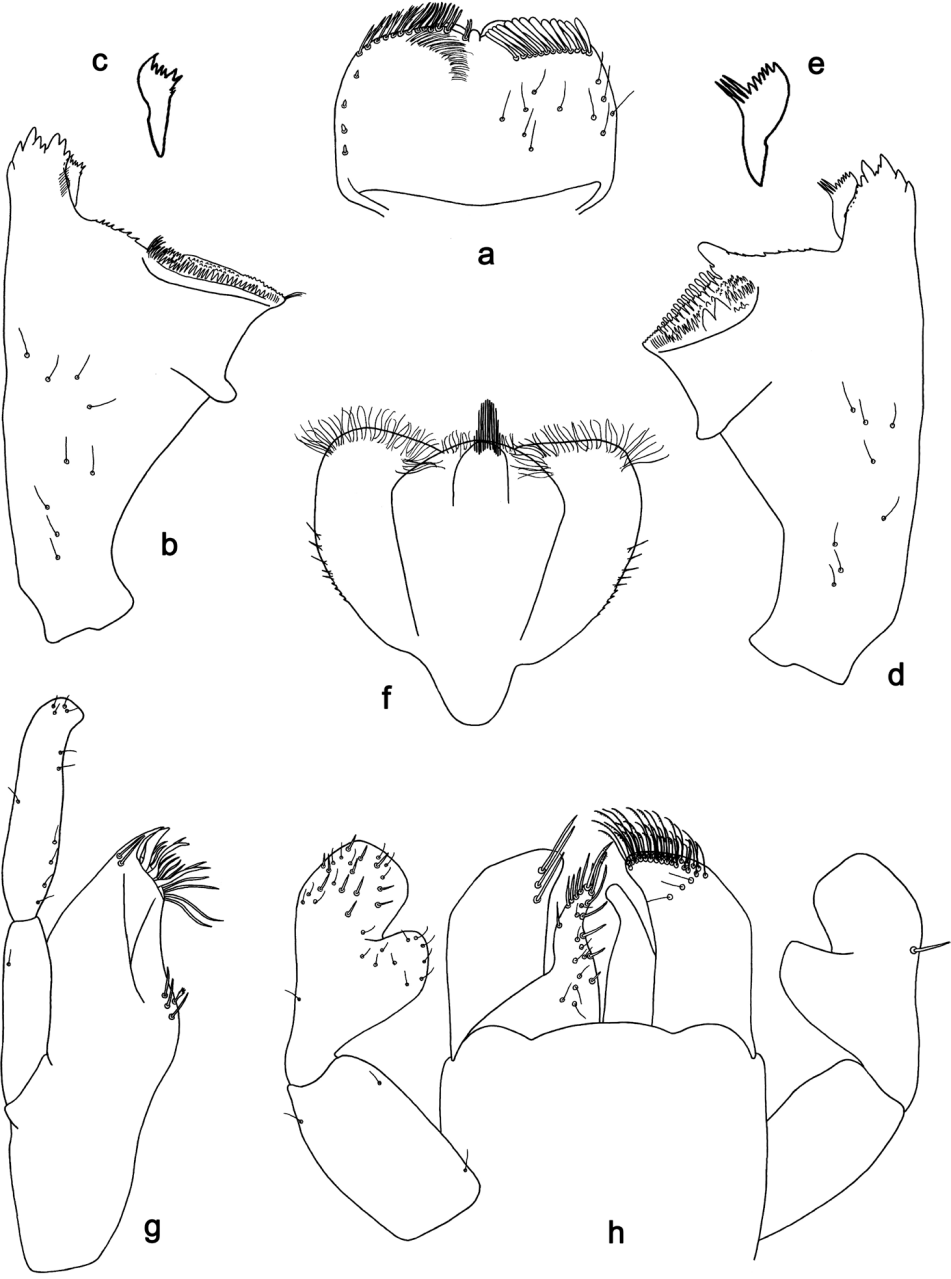
*Labiobaetis bakerae* sp. nov., larva morphology: **a** Labrum **b** Right mandible **c** Right prostheca **d** Left mandible **e** Left prostheca **f**Hypopharynx**g** Maxilla **h** Labium.

**Figure 2.**
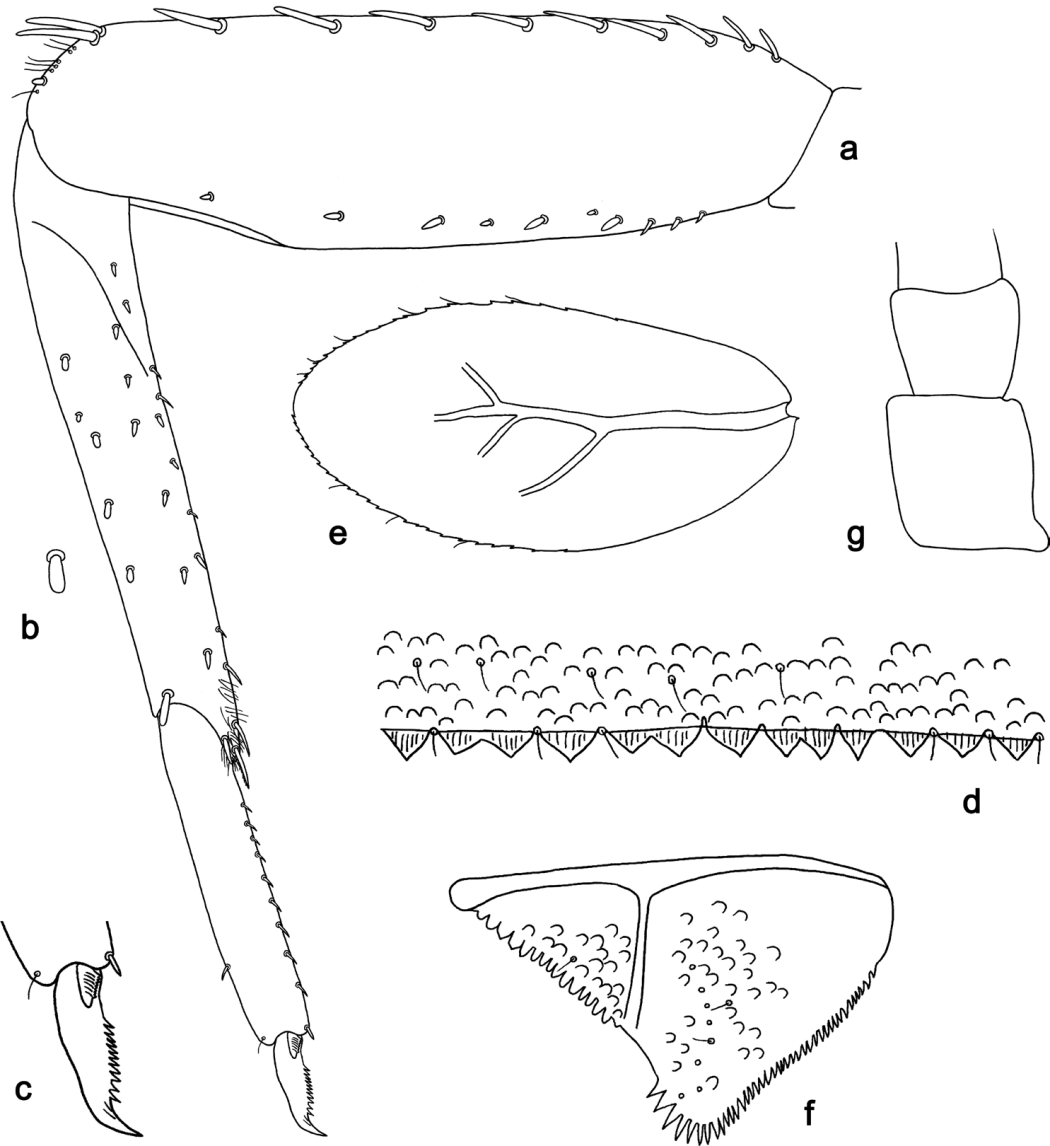
*Labiobaetis bakerae* sp. nov., larva morphology: **a** Foreleg **b** Tibia dorsal seta **c** Fore claw **d** Tergum IV **e** Gill IV **f** Paraproct **g** Antennal scape.

Figures 1 , 2 , 10a , 14 , 15c

###### Diagnosis.

**Larva.** Following combination of characters: A) dorsal surface of labrum with submarginal arc of 13–15 long, clavate setae; B) labial palp segment II with a broad, thumb-like distomedial protuberance, segment III slightly pentagonal; C) left mandible without setae at apex of mola; D) fore femur rather broad, length 3.4× maximum width, dorsal margin with 8–11 curved, spine-like setae; E) paraproct distally expanded, with 34–39 marginal, stout spines.

###### Description.

**Larva** (Figs 1, 2, 10a). Body length 3.5–4.3 mm; antennae and cerci broken.

*Colouration.* Head, thorax and abdomen dorsally brown; head and thorax with bright median, dorsal suture. Head, thorax and abdomen ventrally light brown; femur ecru, with brown dorsal margin and brown ventrodistomedial spot, tibia and tarsus brown, caudal filaments ecru.

*Antenna* (Fig. 2g) with scape and pedicel subcylindrical, with poorly developed distolateral process at scape.

*Labrum* (Fig. 1a). Rectangular, length 0.6× maximum width. Distal margin with medial emargination and a small process. Dorsally with medium, fine, simple setae scattered over surface; submarginal arc of setae composed of 13–15 long, clavate setae. Ventrally with marginal row of setae composed of anterolateral long, feathered setae and medial long, bifid setae; ventral surface with four short, spine-like setae near lateral and anterolateral margin.

*Right mandible* (Fig. 1b, c). Incisors fused. Outer and inner sets of denticles with 4 + 3 denticles and one minute intermediate denticle. Inner margin of innermost denticle with a row of thin setae. Prostheca robust, apically and distolaterally denticulate. Margin between prostheca and mola straight, with minute denticles. Tuft of setae at apex of mola present.

*Left mandible* (Fig. 1d, e). Incisors fused. Outer and inner sets of denticles with 4 + 3 denticles and one minute intermediate denticle. Prostheca robust, apically with small denticles and comb-shaped structure. Margin between prostheca and mola straight, with minute denticles towards subtriangular process. Subtriangular process long and slender, above level of area between prostheca and mola. Denticles of mola apically constricted. Tuft of setae at apex of mola absent.

Both mandibles with lateral margins almost straight. Basal half with fine, simple setae scattered over dorsal surface.

*Hypopharynx* (Fig. 1f). Lingua approx. as long as superlingua. Lingua longer than broad; medial tuft of stout setae well developed; distal half laterally expanded. Superlingua straight; lateral margin rounded; fine, long, simple setae along distal margin.

*Maxilla* (Fig. 1g). Galea-lacinia with two simple, robust apical setae under crown. Inner dorsal row of setae with three denti-setae, distal denti-seta tooth-like, middle and proximal denti-setae slender, bifid and pectinate. Medially with one bipectinate, spine-like seta and 3–4 medium, simple setae. Maxillary palp 1.5× as long as length of galea-lacinia; 2-segmented; palp segment II 1.5× length of segment I; setae on maxillary palp fine and simple, scattered over surface of segments I and II; apex of last segment rounded, with excavation at inner distolateral margin.

*Labium* (Fig. 1h). Glossa basally broad, narrowing toward apex; shorter than paraglossa; inner margin with five spine-like setae increasing in length distally; apex with two long and one medium, robust, pectinate setae; outer margin with five long, spine-like setae; ventral surface with fine, simple, scattered setae. Paraglossa sub-rectangular, curved inward; apex rounded; with three rows of long, robust, distally pectinate setae in apical area and two or three medium, simple setae in anteromedial area; dorsally with a row of three long, spine-like setae near inner margin. Labial palp with segment I 0.8× length of segments II and III combined. Segment I with fine, simple setae along margins. Segment II with broad, thumb-like distomedial protuberance; distomedial protuberance 0.6× width of base of segment III; inner and outer margins with short, fine, simple setae; dorsally with one or two long, spine-like seta near outer margin. Segment III slightly pentagonal; apex rounded; length 1.1× width; ventrally covered with short, spine-like, simple setae and short, fine, simple setae.

*Hindwing pads* absent.

*Foreleg* (Fig. 2a, b, c). Ratio of foreleg segments 1.3:1.0:0.6:0.2. *Femur*. Length ca. 3× maximum width. Dorsal margin with a row of 8–11 curved, spine-like setae; length of setae 0.29× maximum width of femur. Apex rounded; with one pair of curved, spine-like setae, one or a few short stout setae and some fine, simple setae. Stout, lanceolate setae scattered along the ventral margin; femoral patch absent. *Tibia*. Dorsal margin with a row of stout, apically rounded setae, apically one longer, apically rounded seta. Ventral margin with a row of curved, spine-like setae, on apex a few stout, spine-like, partly bipectinate setae and a tuft of fine, simple setae. Anterior surface scattered with stout, lanceolate setae. Patellotibial suture present on basal 1/3 area. *Tarsus*. Dorsal margin almost bare. Ventral margin with a row of curved, spine-like setae. Tarsal claw with one row of 9–11 denticles; distally pointed; with two stripes; subapical setae absent.

*Terga* (Fig. 2d). Surface with rows of U-shaped scale bases and scattered fine, simple, setae. Posterior margin of tergum IV with triangular spines, wider than long.

*Gills* (Fig. 2e). Present on segments II - VII. Margin with small denticles intercalating fine simple setae. Tracheae partly extending from main trunk towards outer and inner margins. Gill IV as long as length of segments V and 1/3 VI combined. Gill VII as long as length of segments VIII and 1/4 IX combined.

*Paraproct* (Fig. 2f). Distally expanded, with 34–39 stout marginal spines. Surface scattered with U-shaped scale bases, fine simple setae and micropores. Cercotractor with small marginal spines.

###### Etymology.

Dedicated to Dr. Kate Baker (University of Exeter, UK), who collected the specimens in Brunei.

###### Distribution.

Brunei (Fig. 15c).

###### Biological aspects.

The specimens were collected in pools of small lowland forest streams at an altitude of 100 m (Fig. 14).

###### Type-material.

***Holotype*.** Larva (on slide, GBIFCH 00592236), Brunei, Temburong District, Ulu Temburong National Park, Belalong River (near field station), 04°33.07'N, 115°09.41'E, 100 m, V. 2014, K. Baker leg. Deposited in MZL. ***Paratypes*.** 2 larva (on slides, GBIFCH 00658097, GBIFCH 00658084), same data as holotype; 5 larvae (on slides, GBIFCH 00592299, GBIFCH 00592296, GBIFCH 00592282, GBIFCH 00284241, GBIFCH 00592298), Brunei, Temburong District, Ulu Temburong National Park, 04°32.77'N, 115°09.52'E, V. 2014, K. Baker leg.; 3 larvae (on slides, GBIFCH 00592297, GBIFCH 00592295, GBIFCH 00592294), Brunei, Temburong District, Ulu Temburong National Park, 04°32.92'N, 115°09.45'E, V. 2014, K. Baker leg. All material deposited in MZL.

##### Labiobaetis penan sp. nov.

Taxon classificationAnimaliaEphemeropteraBaetidae

21BE64F6-6B0B-55AA-BF3A-4CB998A2938E

http://zoobank.org/18DC8E3B-D831-415B-8F97-D3AC264A8931

**Figure 3.**
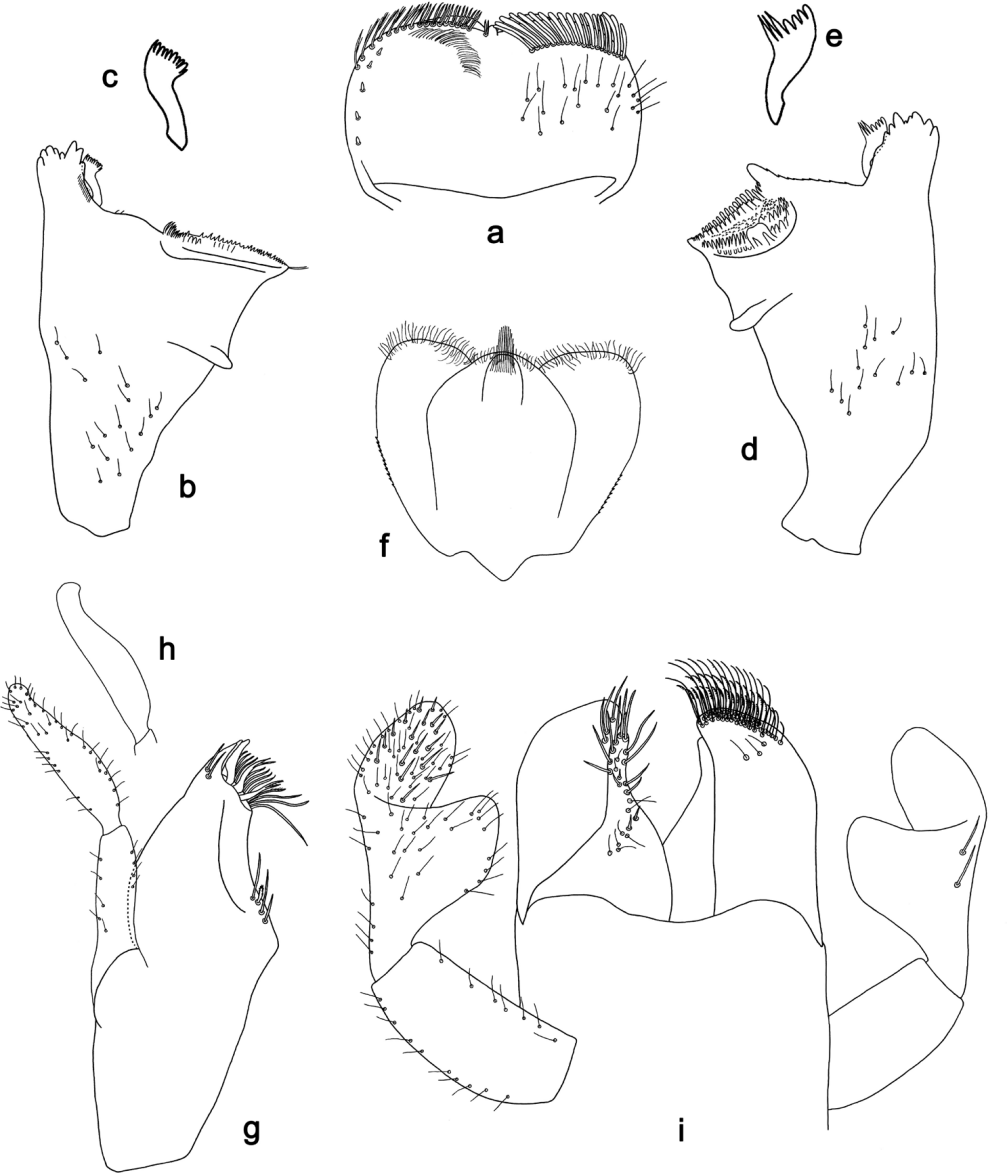
*Labiobaetis penan* sp. nov., larva morphology: **a** Labrum **b** Right mandible **c** Right prostheca **d** Left mandible **e** Left prostheca **f**Hypopharynx**g** Maxilla **h** Labium.

**Figure 4.**
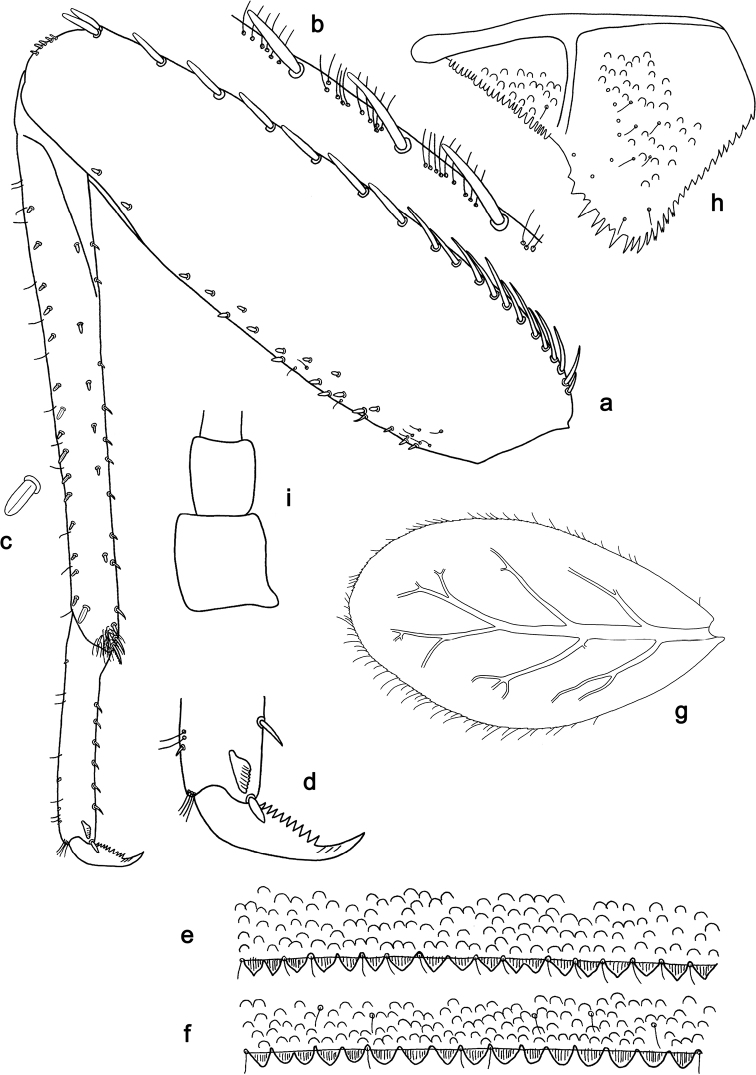
*Labiobaetis penan* sp. nov., larva morphology: **a** Foreleg **b** Femur dorsal setae **c** Tibia dorsal seta **d** Fore claw **e, f** Tergum IV **g** Gill IV **h** Paraproct **i** Antennal scape.

Figures 3 , 4 , 10b , 12a , 13a, c , 15d

###### Diagnosis.

**Larva.** Following combination of characters: A) dorsal surface of labrum with submarginal arc of 18–22 clavate setae; B) labial palp segment II with a broad, thumb-like distomedial protuberance, segment III oblong; C) left mandible without setae at apex of mola; D) fore femur rather broad, length 3.4× maximum width, dorsal margin with a row of 15–19 curved, spine-like setae; E) paraproct distally expanded, with 27–33 marginal, stout spines, some of them with split tips.

###### Description. Larva

(Figs 3, 4, 10b). Body length 3.8–6 mm. Cerci: approx. as long as body length. Terminal filament: approx. as long as 1/2 length of cerci. Antenna: approximately 3× as long as head length.

*Colouration.* Head, thorax, and abdomen dorsally brown; head and thorax with bright, median, dorsal suture. Head, thorax, and abdomen ventrally light brown, legs light brown, caudal filaments light brown.

*Antenna* (Fig. 4i) with scape and pedicel subcylindrical, without distolateral process at scape.

*Labrum* (Fig. 3a). Rectangular, length 0.6× maximum width. Distal margin with medial emargination and a small process. Dorsally with medium, fine, simple setae scattered over surface; submarginal arc of setae composed of 18–22 long, clavate setae. Ventrally with marginal row of setae composed of lateral and anterolateral long, feathered setae and medial long, bifid setae; ventral surface with five short, spine-like setae near lateral and anterolateral margin.

*Right mandible* (Fig. 3b, c). Incisors fused. Outer and inner sets of denticles with 4 + 3 denticles and one minute intermediate denticle. Inner margin of innermost denticle with a row of thin setae. Prostheca robust, apically denticulate. Margin between prostheca and mola slightly convex, with a few minute setae. Tuft of setae at apex of mola present.

*Left mandible* (Fig. 3d, e). Incisors fused. Outer and inner sets of denticles with 4 + 3 denticles and one minute intermediate denticle. Prostheca robust, apically with small denticles and comb-shaped structure. Margin between prostheca and mola straight, with minute denticles towards subtriangular process. Subtriangular process long and slender, above level of area between prostheca and mola. Denticles of mola apically constricted. Tuft of setae at apex of mola absent.

Both mandibles with lateral margins almost straight. Basal half with fine, simple setae scattered over dorsal surface.

*Hypopharynx* (Fig. 3f). Lingua approx. as long as superlingua. Lingua approx. as broad as long; medial tuft of stout setae well developed; distal half not expanded. Superlingua rounded; lateral margin rounded; fine, long, simple setae along distal margin.

*Maxilla* (Fig. 3g, h). Galea-lacinia with two simple, robust apical setae under crown. Inner dorsal row of setae with three denti-setae, distal denti-seta tooth-like, middle and proximal denti-setae slender, bifid and pectinate. Medially with one bipectinate, spine-like seta and three medium, simple setae. Maxillary palp 1.4× as long as length of galea-lacinia; 2-segmented. Palp segment II 1.4× length of segment I. Setae on maxillary palp fine and simple, scattered over surface of segments I and II. Apex of last segment rounded, with strong excavation at inner distolateral margin.

*Labium* (Fig. 3i). Glossa basally broad, narrowing toward apex; shorter than paraglossa; inner margin with five spine-like setae increasing in length distally; apex with two long and one medium, robust, pectinate setae; outer margin with four long, spine-like setae; ventral surface with short, fine, simple and short, spine-like setae. Paraglossa sub-rectangular, curved inward; apex rounded; with three rows of long, robust, distally pectinate setae in apical area and three medium, simple setae in anteromedial area; dorsally with a row of three long, spine-like setae near inner margin. Labial palp with segment I 0,7× length of segments II and III combined. Segment I ventrally with short, fine, simple setae. Segment II with broad, thumb-like distomedial protuberance; distomedial protuberance 1.0× width of base of segment III; inner and outer margin with short, fine, simple setae; dorsally with two long, spine-like, simple setae near outer margin. Segment III oblong; apex rounded; length 1.4× width; ventrally covered with short to medium, spine-like, simple setae and short, fine, simple setae.

*Hindwing pads* absent.

*Foreleg* (Fig. 4a–d). Ratio of foreleg segments 1.1:1.0:0.4:0.1. *Femur*. Length ca. 3× maximum width. Dorsal margin with a row of 15–19 curved, spine-like, apically rounded setae and many long, fine, simple setae and partly a few stout setae near margin; length of setae 0.28× maximum width of femur. Apex rounded; with one pair of curved, spine-like setae and some short, stout setae. Many stout, lanceolate setae scattered along ventral margin; femoral patch poorly developed. *Tibia.* Dorsal margin with a row of stout, lanceolate, apically rounded setae and fine, simple setae; on apex one larger, lanceolate, apically rounded seta. Ventral margin with a row of curved, spine-like setae, on apex one bipectinate, spine-like seta and a tuft of long, fine, simple setae. Anterior surface scattered with stout, lanceolate setae. Patellotibial suture present on basal 1/3 area. *Tarsus.* Dorsal margin with a row of small, stout setae and fine, simple setae. Ventral margin with a row of curved, spine-like setae. Tarsal claw with one row of 9–11 denticles; distally pointed; with three stripes; subapical setae absent.

*Terga* (Fig. 4e, f). Surface with rows of U-shaped scale bases. Posterior margin of tergum IV with triangular or rounded spines, wider than long.

*Gills* (Fig. 4g). Present on segments II - VII. Margin with small denticles intercalating both short and medium, fine, simple setae. Tracheae extending from main trunk to inner and outer margins. Gill IV as long as length of segments V and 1/3 VI combined. Gill VII as long as length of segment VIII.

*Paraproct* (Fig. 4h). Distally expanded, with 27–33 stout marginal spines, some of them with split tips. Surface scattered with U-shaped scale bases, fine, simple setae and micropores. Cercotractor with small marginal spines.

###### Description. Male imago

(Fig. 12a, 13a, c). Body length 3.8 mm, forewing length 4.4 mm.

*Colouration.* Head light beige. Turbinate eyes orange, shaft proximally lighter. Thorax light beige with lateral brown markings (Fig. 13c). Legs light brown. Wings hyaline, venation hyaline. Abdomen dorsally whitish with lateral orange brown markings (Fig. 13c), segment VII dorsally orange brown.

*Forewing* (Fig. 12a). Pterostigma with three cross-veins, distal one bifurcated and reaching subcostal vein, in the middle a short one not reaching subcostal vein and the proximal one reaching subcostal vein; double intercalary veins generally shorter than distance between corresponding main veins at wing margin.

*Hindwing* absent.

*Genitalia* (Fig. 13a). Basal segment of gonostylus (unistyliger) with inner margin apically only slightly expanded; segments I and II almost completely fused; constriction at base of segment II; segment III ovoid. Styliger plate between unistyligers poorly developed, distal margin straight.

###### Etymology.

Dedicated to the indigenous Penan people of Borneo.

###### Distribution.

Indonesia: Kalimantan, Brunei, Malaysia: Sabah (Fig. 15d).

###### Biological aspects.

The specimens were collected in small, shallow forest streams at altitudes from 100 m to 1,450 m, partly in leaf packs.

###### Ontogenetic association.

With genetics, one male imago shares an identical COI sequence with two larvae from the same location (K2P 0%; Table 3).

###### Type-material.

***Holotype*.** Larva (on slide, GBIFCH 00672299), Malaysia, Sabah, creek near Kundasang, sec. forest, 06°00.40'N, 116°32.80'E, 1450 m, 15.III.2008, Mendoza leg., deposited in PNM. ***Paratypes*.** 2 larvae (on slides, GBIFCH 00654918, GBIFCH 00592242), same data as holotype; 2 male imagos (1 in alcohol and wing on slide, GBIFCH 00672296, GBIFCH 00606853, 1 in alcohol, GBIFCH 00515330), same data as holotype. All paratypes deposited in MZL. ***Other material*.** 2 larvae (1 on slide, GBIFCH 00592252, 1 in alcohol, GBIFCH 00658087), Brunei, Temburong District, Ulu Temburong National Park, Belalong River (near field station), 04°32.82'N, 115°09.50'E, 100 m, V. 2014, K. Baker leg.; 1 larva (in alcohol, GBIFCH 00515373), Brunei, Temburong District, Ulu Temburong National Park, Sungai Mata Ikan (tributary to Belalong River, small creek near station), 04°32.83'N, 115°09.38'E, 110 m, V. 2014, K. Baker leg.; 5 larvae (1 on slide, GBIFCH 00592239, 4 in alcohol, GBIFCH 00515327), Brunei, Temburong District, Ulu Temburong National Park, Belalong River tributary, 04°32.63'N, 115°08.85'E, 170 m, V. 2014, K. Baker leg.; 1 larva (on slide, GBIFCH 00592286), Indonesia, East Kalimantan, Bas. Malinau, River Rian, loc. Langap South (1999-block 24), tributary, 03°01.67'N, 116°31.08'E, 11.VII.2000, P. Derleth leg.; 20 larvae (1 on slide, GBIFCH 00592283, 19 in alcohol, GBIFCH 00515396, GBIFCH 515385, GBIFCH 00515386, GBIFCH 00515388, GBIFCH 00515294, GBIFCH 00515314), Indonesia, East Kalimantan, Bas. Malinau, River Rian, loc. Langap South (1997-block 6), trib. Belakau, 03°04.07'N, 116°30.43'E, 05.VII.2000, P. Derleth leg.; 3 larvae (1 on slide, GBIFCH 592284, 2 in alcohol, GBIFCH 00515397), Indonesia, East Kalimantan, Bas. Malinau, River Seturan, loc. Seturan (2001-block 57), trib. Tamalang, 10.IV.2001, P. Derleth leg.; 18 larvae (1 on slide, GBIFCH 00592251, 17 in alcohol, GBIFCH 00515304, GBIFCH 00515390, GBIFCH 00515302, GBIFCH 00515387), Indonesia, East Kalimantan, Bas. Malinau, River Seturan, loc. Seturan (2001-block 57), trib. Bengahau, 02°59.37'N, 116°30.77'E, 08.VIII.2000, P. Derleth leg., 1 larva (in alcohol, GBIFCH 00515389), Indonesia, East Kalimantan, Bas. Malinau, River Seturan, loc. Seturan (1999-block 39-40), trib. Temalat (Sungai Guang), 03°00.17'N, 116°32.40'E, 01.VII.2000, P. Derleth leg.; 9 larvae (in alcohol, GBIFCH 00515305), Indonesia, East Kalimantan, Bas. Malinau, River Rian, Langap South (1995), trib. Ngayo, 03°04.93'N, 116°30.97'E, 13.VII.2000, P. Derleth leg.; 8 larvae (in alcohol, GBIFCH 515301), Indonesia, East Kalimantan, Bas. Malinau, River Seturan, loc. Seturan (2001-block 57), trib. Tamalang, 19.VII.2000, P. Derleth and F. Béboux leg.; 2 larvae (in alcohol, GBIFCH 00515320), Indonesia, East Kalimantan, Bas. Malinau, River Seturan, loc. Seturan (2000-block 45), trib. Wok (Sungai Guang), 03°00.15'N, 116°32.42'E, 29.VI.2000, P. Derleth leg.; 7 larvae (in alcohol, GBIFCH 00515293), Indonesia, East Kalimantan, Bas. Malinau, River Seturan, loc. Seturan (2000-block 44-45), trib. Wok (Sungai Guang), 02°59.20’’N 116°33.18'E, 17.VI.2000, P. Derleth and J.-L. Gattolliat leg.; 1 larva (in alcohol, GBIFCH 00515318), Indonesia, East Kalimantan, Bas. Malinau, River Seturan, loc. Seturan (2000-block 44-45), trib. Wok (Sungai Guang), 02°59.20'N, 116°33.18'E, 16.VI.2000, P. Derleth and J.-L. Gattolliat leg.; 13 larvae (in alcohol, GBIFCH 00515300, GBIFCH 00515298, GBIFCH 00515311, GBIFCH 00515295), Indonesia, East Kalimantan, Bas. Malinau, River Seturan, loc. Seturan (2000-block 43), trib. Temalat (Sungai Guang), 02°59.48'N, 116°33.48'E, 16.VIII.2000, P. Derleth and R. Schlaepfer leg.; 1 larva (in alcohol, GBIFCH 00515384), Indonesia, East Kalimantan, Bas. Malinau, River Seturan, loc. Seturan (1998-block 28), trib. Kipah, 03°01.80'N, 116°01.80'E, 29.III.2001, P. Derleth leg.; 2 larvae (1 on slide, GBIFCH 00592287, 1 in alcohol, GBIFCH 00515319), Indonesia, East Kalimantan, Bas. Malinau, River Rian, loc. Seturan (1998-block 32-33), tributary, 03°00.95'N, 116°32.27'E, 23.VI.2000, P. Derleth and J.-L. Gattolliat leg.; 4 larvae (in alcohol, GBIFCH 00515303), Indonesia, East Kalimantan, Bas. Malinau, River Seturan, loc. Seturan (1999-block 27), tributary, 03°00.95'N, 116°30.52'E, 10.VII.2000, P. Derleth leg. All material deposited in MZL.

### *Labiobaetis operosus* group of species (Kaltenbach and Gattolliat 2019)

Following combination of characters: A) dorsal surface of labrum with submarginal arc of feathered setae; B) labial palp segment II with thumb-like or lobed distomedial protuberance; C) seven pairs of gills; D) hindwing pads well developed; E) distolateral process at scape well developed.

#### Labiobaetis dayakorum sp. nov.

Taxon classificationAnimaliaEphemeropteraBaetidae

DB83AAA0-FC2E-594B-A767-F8339E6624A0

http://zoobank.org/A0B3DDF0-8270-4379-9BD8-D0CE90D43EE3

**Figure 5.**
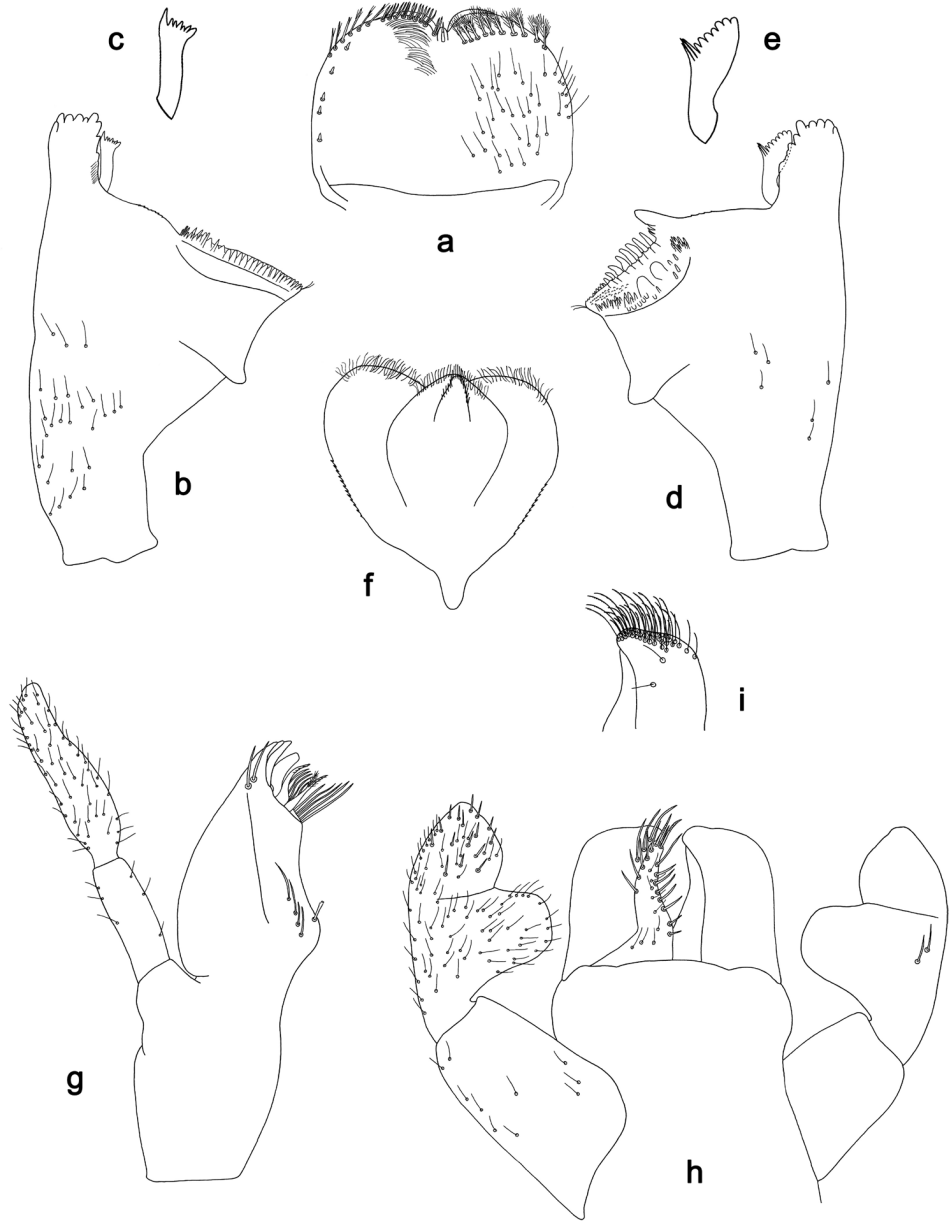
*Labiobaetis dayakorum* sp. nov., larva morphology: **a** Labrum **b** Right mandible **c** Right prostheca **d** Left mandible **e** Left prostheca **f**Hypopharynx**g** Maxilla **h** Labium **i** Apex of paraglossa.

**Figure 6.**
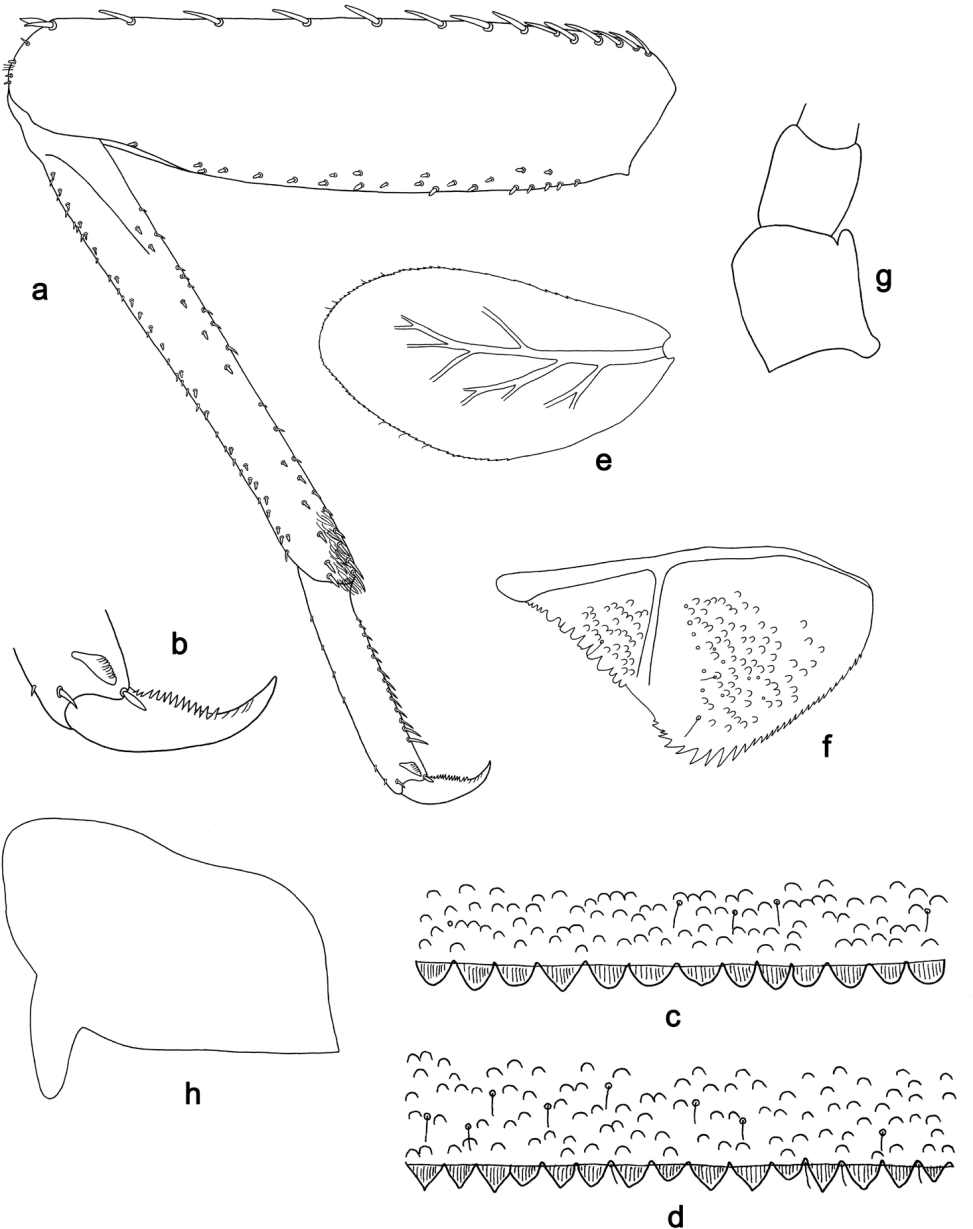
*Labiobaetis dayakorum* sp. nov., larva morphology: **a** Foreleg **b** Fore claw **c, d** Tergum IV **e** Gill IV **f** Paraproct **g** Antennal scape **h** Metanotum.

Figures 5 , 6 , 11a , 15c

##### Diagnosis.

**Larva.** Following combination of characters: A) dorsal surface of labrum with submarginal arc of 10–12 long, feathered setae; B) labial palp segment II with a large, lobed distomedial protuberance, segment III slightly pentagonal; C) fore femur rather broad, length ca. 4× maximum width, dorsal margin with a row of 12–14 curved, spine-like setae; D) hindwing pads well developed; E) paraproct distally not expanded, with 30–37 marginal, stout spines.

##### Description.

**Larva** (Figs 5, 6, 11a). Body length 5.2 mm; antenna: approximately 2.5× as long as head length; cerci broken.

*Colouration.* Head, thorax and abdomen dorsally brown; head and thorax with bright median, dorsal suture, abdominal segment X light brown. Head, thorax and abdomen ventrally light brown, legs light brown with a brown spot medially and apically on femur, caudal filaments light brown.

*Antenna* (Fig. 6g) with scape and pedicel subcylindrical, with well-developed distolateral process at scape.

*Labrum* (Fig. 5a). Rectangular, length 0.7× maximum width. Distal margin with medial emargination and a small process. Dorsally with medium to long, fine, simple setae scattered over surface; submarginal arc of setae composed of 10–12 long, feathered setae. Ventrally with marginal row of setae composed of anterolateral long, feathered setae and medial long, bifid setae; ventral surface with five short, spine-like setae near lateral and anterolateral margin.

*Right mandible* (Fig. 5b, c). Incisors fused. Outer and inner sets of denticles with 4 + 3 denticles and one minute intermediate denticle. Inner margin of innermost denticle with a row of thin setae. Prostheca robust, apically denticulate. Margin between prostheca and mola slightly convex, with minute denticles. Tuft of setae at apex of mola present.

*Left mandible* (Fig. 5d, e). Incisors fused. Outer and inner sets of denticles with 4 + 3 denticles and one minute intermediate denticle. Prostheca robust, apically with small denticles and comb-shaped structure. Margin between prostheca and mola straight, with minute denticles towards subtriangular process. Subtriangular process long and slender, above level of area between prostheca and mola. Denticles of mola apically constricted. Tuft of setae at apex of mola present.

Both mandibles with lateral margins almost straight. Basal half with fine, simple setae scattered over dorsal surface.

*Hypopharynx* (Fig. 5f). Lingua approx. as long as superlingua. Lingua longer than broad; medial tuft of stout setae poorly developed; distal half laterally expanded. Superlingua rounded; lateral margin rounded; fine, long, simple setae along distal margin.

*Maxilla* (Fig. 5g). Galea-lacinia with two simple, robust apical seta under crown. Inner dorsal row of setae with three denti-setae, distal denti-seta tooth-like, middle and proximal denti-setae slender, bifid and pectinate. Medially with one bipectinate, spine-like seta and four medium, simple setae. Maxillary palp 1.2× as long as length of galea-lacinia; 2-segmented; palp segment II 1.6× length of segment I; setae on maxillary palp fine and simple, scattered over surface of segments I and II; apex of last segment rounded, with excavation at inner distolateral margin.

*Labium* (Fig. 5h, i). Glossa basally broad, narrowing toward apex; shorter than paraglossa; inner margin with seven or eight spine-like setae increasing in length distally; apex with two long and one medium, robust, pectinate setae; outer margin with five or six long, spine-like setae; ventral surface with short, fine, simple, scattered setae. Paraglossa sub-rectangular, curved inward; apex rounded; with three rows of long, robust, distally pectinate setae in apical area and two medium, simple setae in anteromedial area; dorsally with a row of three long, spine-like setae near inner margin. Labial palp with segment I 0.9× length of segments II and III combined. Segment I ventrally with short, fine, simple setae. Segment II with large, lobed distomedial protuberance; distomedial protuberance 0.7× width of base of segment III; inner and outer margin with short, fine, simple setae; dorsally with two medium, spine-like, simple setae near outer margin. Segment III slightly pentagonal; apex truncate; length 1.1× width; ventrally covered with short, spine-like, simple setae and short, fine, simple setae.

*Hindwing pads* (Fig. 6h) well developed.

*Foreleg* (Fig. 6a, b). Ratio of foreleg segments 1.1:1.0:0.4:0.2. *Femur*. Length ca. 4× maximum width. Dorsal margin with a row of 12–14 curved, spine-like setae; length of setae 0.26× maximum width of femur. Apex rounded, with one pair of curved, spine-like setae and some short, stout setae. Many short, stout, lanceolate setae scattered along the ventral margin; femoral patch absent. *Tibia.* Dorsal margin with a row of short, stout setae, on apex one longer seta, and a row of short, stout setae close to dorsal margin. Ventral margin with a row of curved, spine-like setae, on apex two spine-like seta and a tuft of long, fine, simple setae. Anterior surface scattered with stout, lanceolate setae. Patellotibial suture present on basal 1/3 area. *Tarsus.* Dorsal margin with a row of short, stout setae. Ventral margin with a row of curved, spine-like setae. Tarsal claw with one row of 9–13 denticles; distally pointed; with four stripes; subapical setae absent.

*Terga* (Fig. 6c, d). Surface with irregular rows of U-shaped scale bases and scattered fine, simple setae. Posterior margin of tergum IV with rounded or triangular spines, wider than long.

*Gills* (Fig. 6e). Present on segments I - VII. Margin with small denticles intercalating fine simple setae. Tracheae extending from main trunk to inner and outer margins. Gill I as long as length of ½ segment II. Gill IV as long as length of segments V and 1/3 VI combined. Gill VII as long as length of segments VIII and 1/3 IX combined.

*Paraproct* (Fig. 6f). Distally not expanded with 30–37 stout marginal spines. Surface scattered with U-shaped scale bases, fine, simple setae and micropores. Cercotractor with medium marginal spines.

##### Etymology.

Dedicated to the indigenous Dayak people of Borneo.

##### Distribution.

Indonesia: Kalimantan (Fig. 15c).

##### Biological aspects.

The specimens were collected at an altitude of 200 m, partly in a large river.

##### Type-material.

**Holotype.** Larva (on slide, GBIFCH 00592281), Indonesia, East Kalimantan, Bas. Malinau, River Seturan, loc. Seturan, tributary, 03°00.08'N, 116°30.80'E, 28.III.2001, P. Derleth and B. Feldmeyer leg. **Paratypes.** 1 larva (on slide, GBIFCH 00592255), Indonesia, East Kalimantan, Bas. Malinau, River Rian, loc. Seturan (1998-block 32-33), tributary, 03°00.95'N, 116°32.27'E, 30.III.2001, P. Derleth leg.; 1 larva (on slide, GBIFCH 00592256), Indonesia, East Kalimantan, Bas. Malinau, River Seturan, loc. Seturan (2001-block 57), trib. Benganau, 02°59.37'N, 116°30.77'E, 11.IV.2001, P. Derleth and B. Feldmeyer leg. All material deposited in MZL.

### Not assigned to a group

#### Labiobaetis borneoensis

Taxon classificationAnimaliaEphemeropteraBaetidae

(Müller-Liebenau, 1984)

5DF31DA7-EE10-5AEE-9D71-458B922A3965

Figures 8 , 10c , 12b , 13b , 15b

##### Diagnosis.

**Larva.** Following combination of characters: A) dorsal surface of labrum with submarginal arc of 9–10 feathered setae (Müller-Liebenau 1984b: fig. 2a); B) labial palp segment II with a large, lobed distomedial protuberance, segment III oblong, apically slightly pointed; C) fore femur rather slender, length 3.6× maximum width, dorsal margin with a row of 11-13 curved, spine-like setae (Müller-Liebenau 1984b: fig. 2i); D) seven pairs of gills; E) hindwing pads present, small; F) distolateral process at scape well developed (Müller-Liebenau 1984b: fig. 2f).

##### Description.

**Male imago** (Fig. 12b, 13b). Body length 4.6 mm, forewing length 4.5 mm.

*Colouration.* Head beige. Turbinate eyes dark orange brown, shaft slightly lighter. Thorax beige, pronotum dark olive brown, mesonotum olive. Wings hyaline, venation hyaline. Abdomen: terga olive, sterna transparent.

*Forewing* (Fig. 12b). Pterostigma with seven cross-veins, only two proximal ones reaching subcostal vein; double intercalary veins shorter than distance between corresponding main veins at wing margin.

*Genitalia* (Fig. 13b). Basal segment of gonostylus (unistyliger) with inner margin apically slightly expanded; segments I and II almost completely fused; constriction at base of segment II; segment III quadrangular. Styliger plate between unistyligers trapezoidal, distal margin slightly concave.

##### Distribution.

Indonesia: Kalimantan, Malaysia: Sabah, Brunei (Fig. 15b).

##### Biological aspects.

The specimens were collected at altitudes between 100 m to 300 m, partly on bottom gravel, rock surface or submerged wood in stream run or riffles.

##### Ontogenetic association.

With genetics, one male imago shares an identical COI sequence with a larva from the same location (K2P 0%, Table 3).

**Figure 7.**
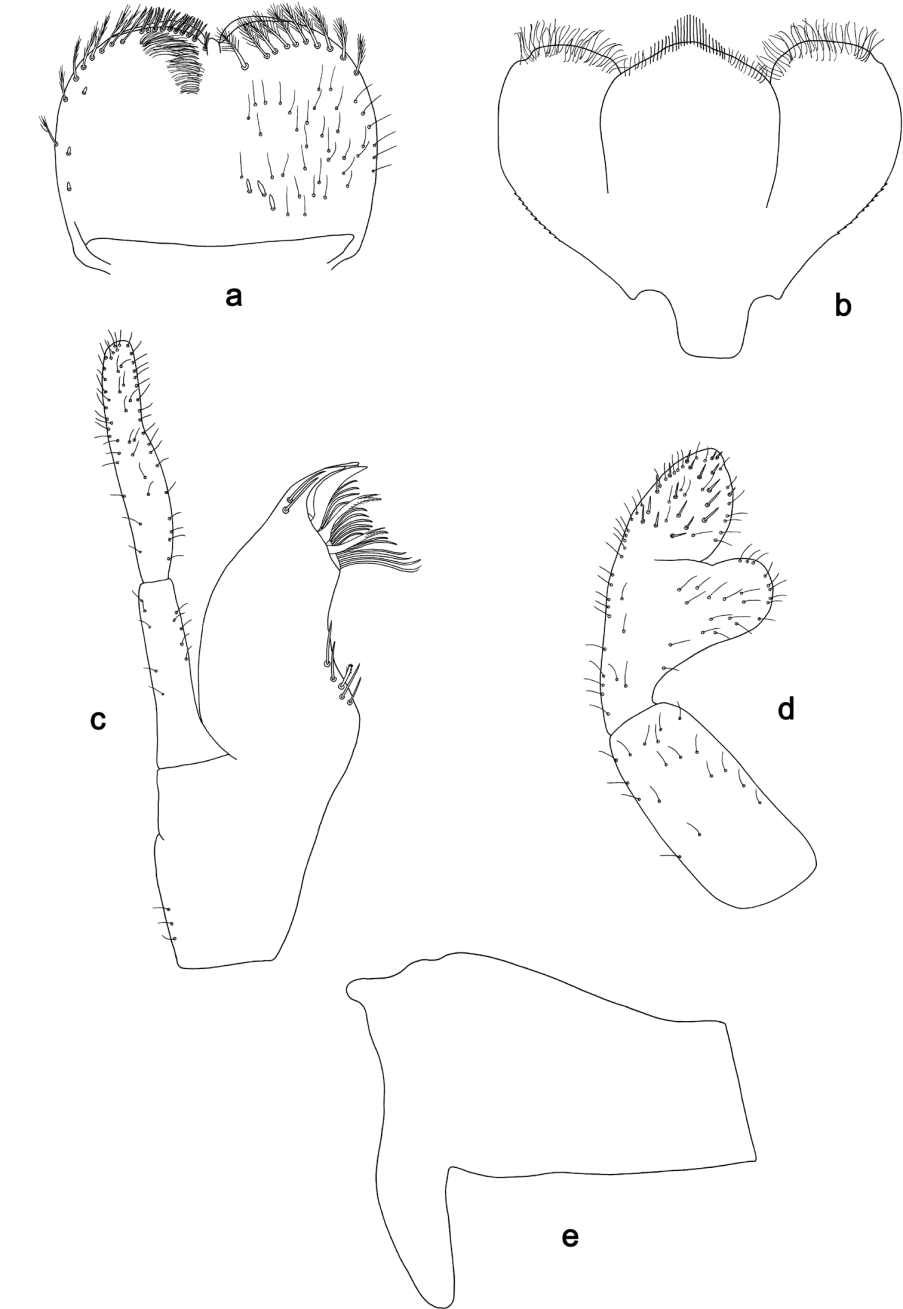
*Labiobaetis paraoperosus*, larva morphology: **a** Labrum **b**Hypopharynx**c** Maxilla **d** Labial palp **e** Metanotum.

**Figure 8.**
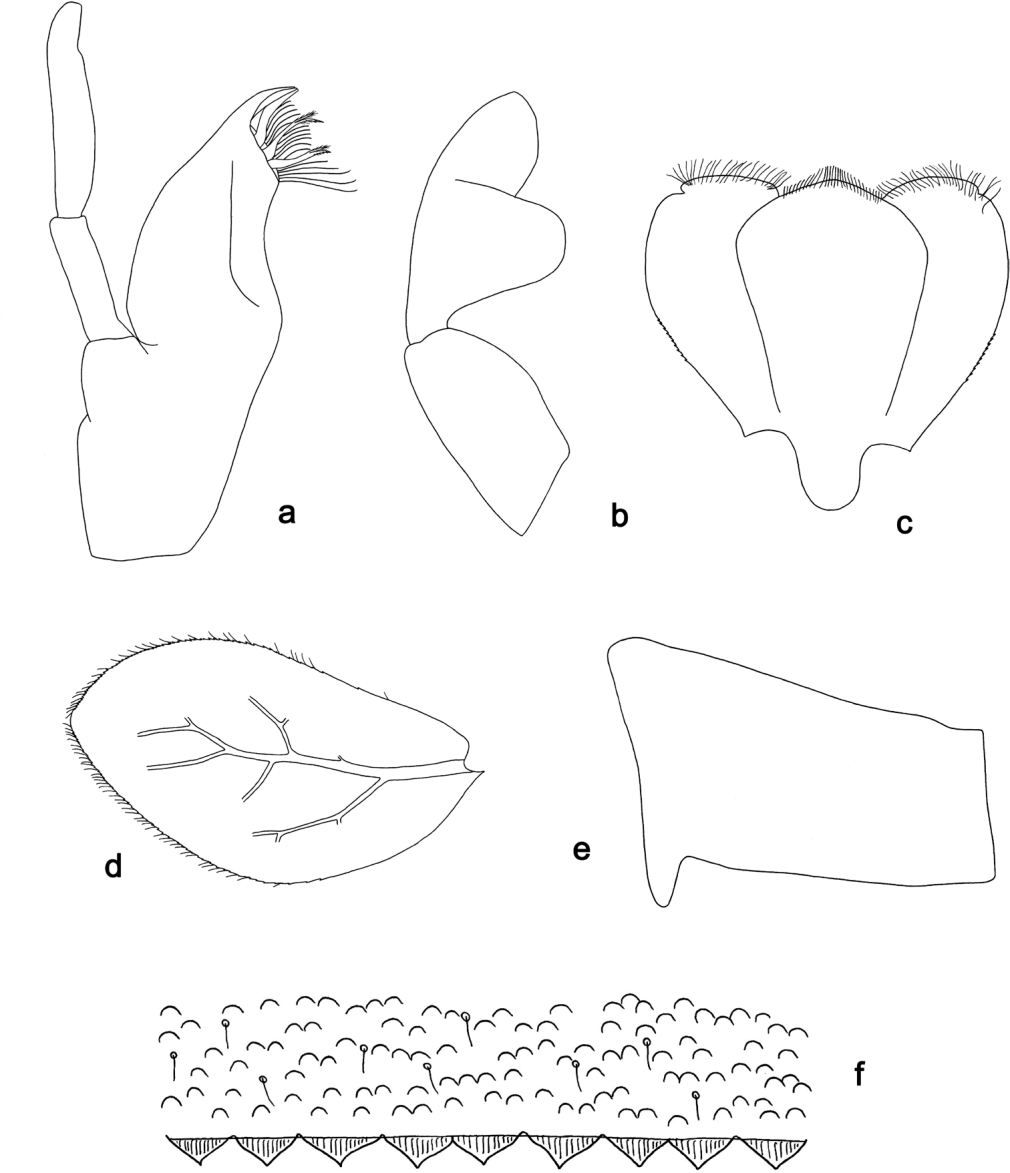
*Labiobaetis borneoensis*, larva morphology: **a** Maxilla **b** Labial palp **c**Hypopharynx**d** Gill IV **e** Metanotum **f** Tergum IV.

##### Examined material.

11 larvae (2 on slides, GBIFCH 00592240, GBIFCH 00658085, 9 in alcohol, GBIFCH 00515368, GBIFCH 00515370), Brunei, Temburong District, Ulu Temburong National Park, Belalong River (near field station), 04°32.82'N, 115°09.50'E, 100 m, V. 2014, K. Baker leg.; 1 larva (in alcohol, GBIFCH 00515369), Brunei, Temburong District, Ulu Temburong National Park, Sungai Seluju (small tributary to Temburong River, near station), 04°33.83'N, 115°08.92'E, 90 m, V. 2014, K. Baker leg.; 3 larvae (2 on slides, GBIFCH 00658081, GBIFCH 00592244, 1 in alcohol, GBIFCH 00515372), Malaysia, Sabah, Tawau River, primary forest, 04°24.08'N, 117°53.35'E, 280 m, 12.III.2008, Mendoza leg.; 1 male imago (in alcohol and wing on slide, GBIFCH 00672289, GBIFCH 00606854), Malaysia, Sabah, Tawau River, primary forest, 04°24.08'N, 117°53.35'E, 280 m, 12.III.2008, Mendoza leg.; 9 larvae (1 on slide, GBIFCH00465236, 8 in alcohol, GBIFCH 00515394, GBIFCH 00515309, GBIFCH 00515296, GBIFCH 00515376, GBIFCH 00515299), Indonesia, East Kalimantan, Bas. Malinau, River Rian, loc. Langap South (1997-bloc 6), trib. Belakau, 03°04.07'N, 116°30.43'E, 07.VII.2000, P. Derleth leg.; 14 larvae (in alcohol, GBIFCH 00515392, GBIFCH 00515393, GBIFCH 515315, GBIFCH 515312, GBIFCH 515306), Indonesia, East Kalimantan, Bas. Malinau, River Seturan, loc. Seturan (2001-bloc 57), trib. Bengahau, 02°59.37'N, 116°30.77‘E, 08.VIII.2000, P. Derleth leg.; 6 larvae (in alcohol, GBIFCH 00515317, GBIFCH 00515321, GBIFCH 00515383), Indonesia, East Kalimantan, Bas. Malinau, River Seturan, loc. Seturan (1999-block 39-40), trib. Temalat (Sungai Guang), 03°00.17'N, 116°32.40'E, 01.VII.2000, P. Derleth leg.; 1 larva (in alcohol, GBIFCH 00515313), Indonesia, East Kalimantan, Bas. Malinau, River Rian, loc. Langap South (1995), trib. Ngayo, 03°01.80'N, 116°29.80‘E, 08.VII.2000, P. Derleth leg.; 2 larvae (in alcohol, GBIFCH 00515382, GBIFCH 00515310), Indonesia, East Kalimantan, Bas. Malinau, River Rian, Langap South (1995), trib. Ngayo, 03°04.93'N, 116°30.97'E, 13.VII.2000, P. Derleth leg.; 3 larvae (in alcohol, GBIFCH 00515395, GBIFCH 00515297, GBIFCH 00515378), Indonesia, East Kalimantan, Bas. Malinau, River Seturan, loc. Seturan (2000-block 43), trib. Temalat (Sungai Guang), 02°59.48'N, 116°33.48'E, 16.VIII.2000, P. Derleth and R. Schlaepfer leg.; 3 larvae (1 on slide, GBIFCH00465237, 2 in alcohol, GBIFCH 00515307, GBIFCH 00515375), Indonesia, East Kalimantan, Bas. Malinau, River Rian, loc. Seturan (1998-block 32-33), tributary, 03°00.95'N, 116°32.27'E, 30.III.2001, P. Derleth leg.; 1 larva (in alcohol, GBIFCH 00515316), Indonesia, East Kalimantan, Bas. Malinau, River Seturan, loc. Seturan (1999-block 27), tributary, 03°00.95'N, 116°30.52'E, 10.VII.2000, P. Derleth leg.; 4 larvae (in alcohol, GBIFCH 00515322, GBIFCH 00515377, GBIFCH 515379), Indonesia, East Kalimantan, Bas. Malinau, River Rian, loc. Langap South (1999-block 24), tributary, 03°01.67'N, 116°31.08'E, 11.VII.2000, P. Derleth leg.; 3 larvae (in alcohol, GBIFCH 00515380, GBIFCH 00515381), Indonesia, East Kalimantan, Bas. Malinau, River Seturan, loc. Seturan, main river, 03°00.08'N, 116°30.80'E, 28.III.2001, P. Derleth and B. Feldmeyer leg.; 5 larvae (in alcohol, GBIFCH 00515374), Indonesia, East Kalimantan, Bas. Malinau, River Seturan, loc. Seturan, tributary, 02°59.82'N, 116°31.37'E, 27.IV.2001, P. Derleth and M. Sartori leg. All material deposited in MZL.

#### Labiobaetis moriharai

Taxon classificationAnimaliaEphemeropteraBaetidae

(Müller-Liebenau, 1984)

CD22467E-C6F0-5D53-BDCD-EA5B6307E20B

**Figure 9.**
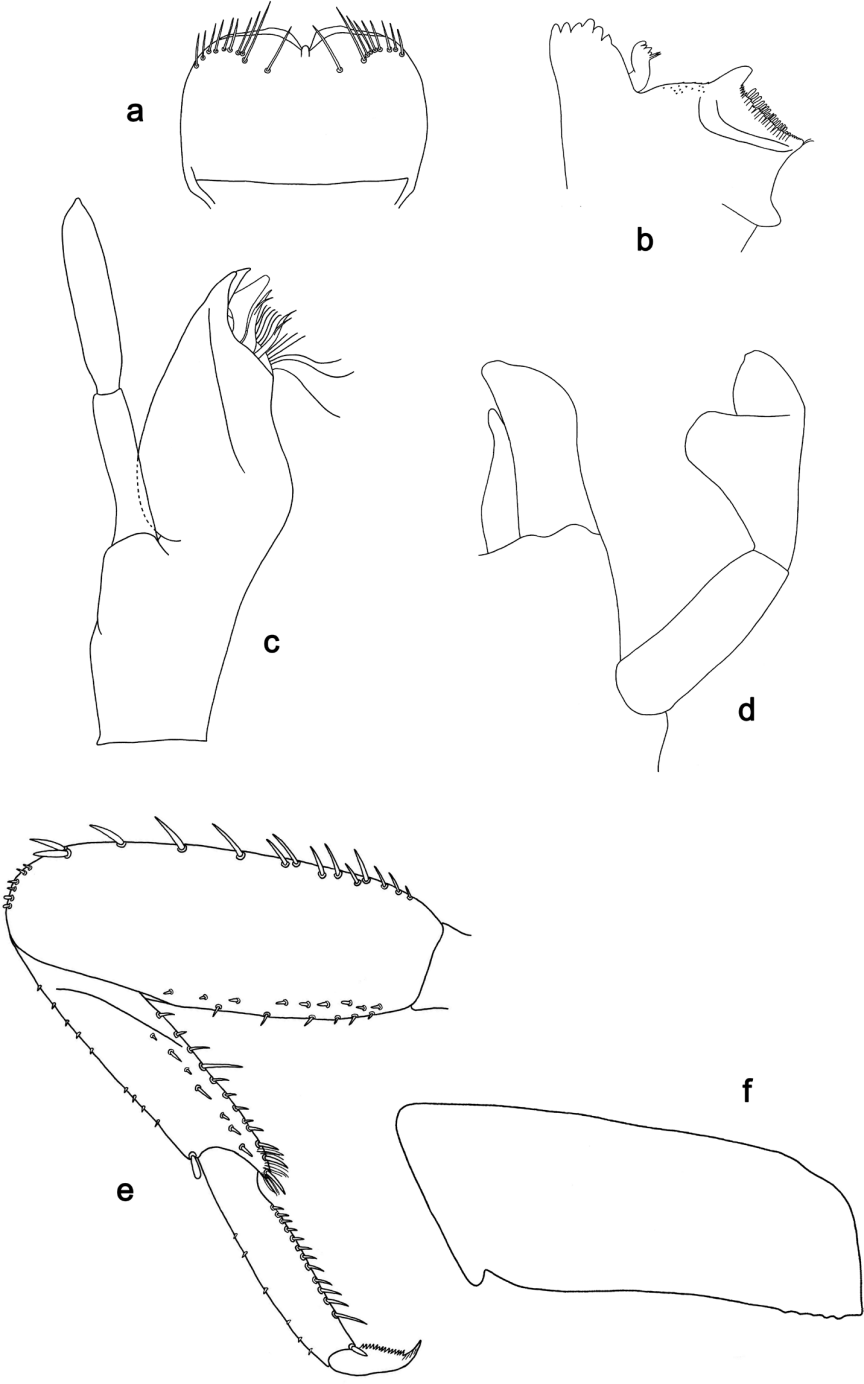
*Labiobaetis moriharai*, larva morphology: **a** Labrum **b** Left mandible **c** Maxilla **d** Labium **e** Foreleg **f** Metanotum.

Figures 9 , 11c , 15a

##### Diagnosis.

**Larva.** Following combination of characters: A) dorsal surface of labrum with submarginal arc of 1 + 8–10 simple setae, the first three after central seta longer than others and decreasing in length; B) labial palp segment II with a large, lobed distomedial protuberance, segment III conical, apically slightly truncate; C) fore femur rather broad, length 3.4× maximum width, dorsal margin with a row of ca. 10 curved, spine-like setae; D) six pairs of gills; E) hindwing pads present, minute; F) scape with well-developed distolateral process (Müller-Liebenau 1984a: fig. 10f); G) paraproct distally not expanded, with ca. 12 stout marginal spines (Müller-Liebenau 1984a: fig. 10l).

**Figure 10.**
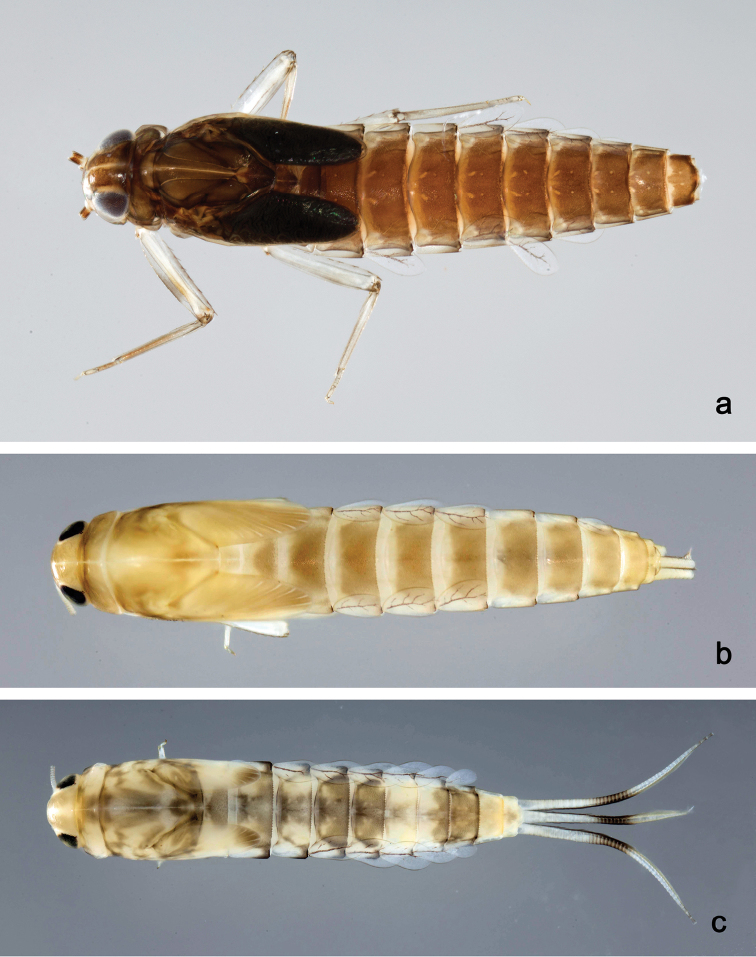
Habitus, larvae, dorsal view: **a***Labiobaetis bakerae* sp. nov. **b***Labiobaetis penan* sp. nov. **c***Labiobaetis borneoensis*.

##### Distribution.

Malaysia: Selangor, Sabah; Vietnam; Brunei (Fig. 15a).

##### Biological aspects.

The specimens were collected at altitudes from 100 m to 300 m, partly on bottom gravel, rock surface or vegetation in stream run or riffles.

##### Examined material.

***Paratype*.** 1 larva (on slide, no. 41), W. Malaysia, Trib. of Gombak River, 16 ½ miles N of Kuala Lumpur, 14.XI.[19]68, Coll. Bishop. ***Other material*.** 1 larva (on slide, GBIFCH 00658106), Brunei, Temburong District, Ulu Temburong National Park, Belalong River (near field station), 04°32.82'N, 115°09.50'E, 100 m, V. 2014, K. Baker leg.; 1 larva (on slide, GBIFCH 00592243), Brunei, Temburong District, Ulu Temborong National Park, Belalong River tributary, 04°32.63'N, 115°08.85'E, 170 m, V. 2014, K. Baker leg.; 5 larvae (2 on slides, GBIFCH 00592241, GBIFCH 00658112, 3 in alcohol, GBIFCH 00515325), Malaysia, Sabah, Tawau River, primary forest, 04°24.23'N, 117°53.58'E, 280 m, 12.III.2008, Mendoza leg. All material deposited in MZL, except paratype in Zoologische Staatssammlung München (ZSM).

### Key to the *Labiobaetis* species of Borneo (larvae)

**Table:** 

1	Dorsal surface of labrum with submarginal arc of clavate setae; hindwing pads absent	**2**
–	Dorsal surface of labrum with submarginal arc of simple or feathered setae; hindwing pads present	**3**
2	Dorsal surface of labrum with submarginal arc of 13–15 setae; 8–11 setae on dorsal margin of femur; gills margin serrated with small denticles and with medium fine, simple setae	***L. bakerae* sp. nov.**
–	Dorsal surface of labrum with submarginal arc of 18–22 setae; 15–19 setae on dorsal margin of femur; gills margin serrated with small denticles and with both short and medium, fine, simple setae	***L. penan* sp. nov.**
3	Dorsal surface of labrum with submarginal arc of simple setae; hindwing pads minute (Fig. 9f)	***L. moriharai***
–	Dorsal surface of labrum with submarginal arc of feathered setae	**4**
4	Hindwing pads small (Fig. 8e)	***L. borneoensis***
–	Hindwing pads well developed (Fig. 6h)	***L. dayakorum* sp. nov.**

### Distribution

The material treated in this study was collected in ca. 20 localities in Borneo, which belong to four different areas, one area in Brunei, two in Sabah (Malaysia), and one in Kalimantan (Indonesia) (Fig. 15). There are still many regions in Borneo as well as in Southeast Asia in general where no sampling of mayflies has yet been done and many species known to date are from a single population only. This implies that the diversity and the distribution must be considered as very preliminary. However, the distribution of the *Labiobaetis* species seems to be very diverse. *Labiobaetis moriharai* has a large distribution (continental and insular) and the other species are endemic to Borneo (Fig. 15). In terms of altitude, the *Labiobaetis* species of Borneo were found from sea level to mountain areas up to 1,450 m. The GPS coordinates of the locations of examined material are given in Table 2.

**Figure 11.**
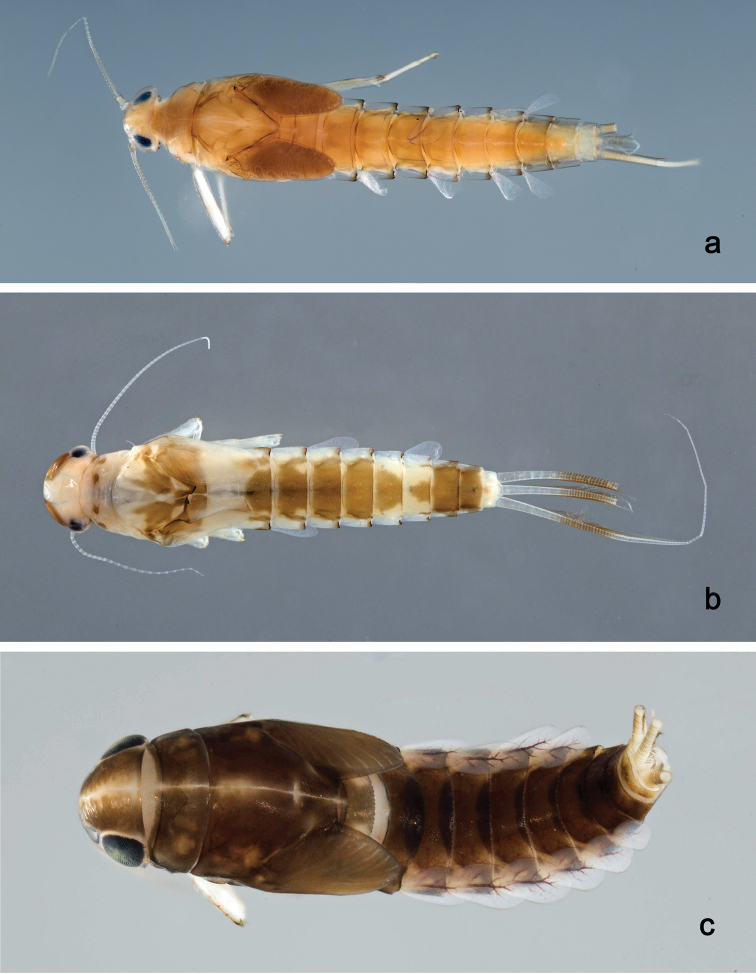
Habitus, larvae, dorsal view: **a***Labiobaetis dayakorum* sp. nov. **b***Labiobaetis paraoperosus***c***Labiobaetis moriharai*.

**Figure 12.**
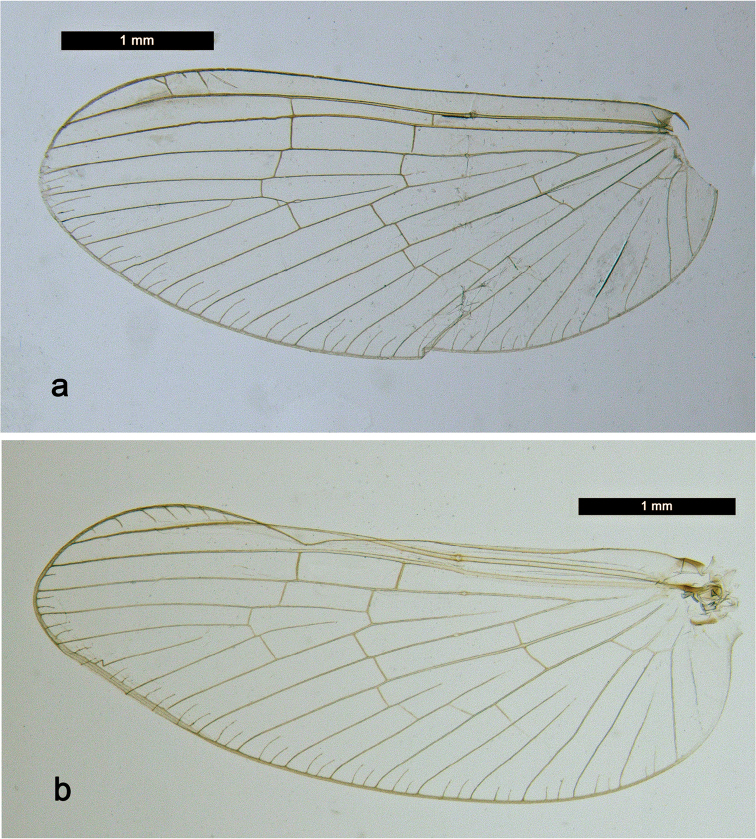
Male imagos, forewings: **a***Labiobaetis penan* sp. nov. **b***Labiobaetis borneoensis*.

**Figure 13.**
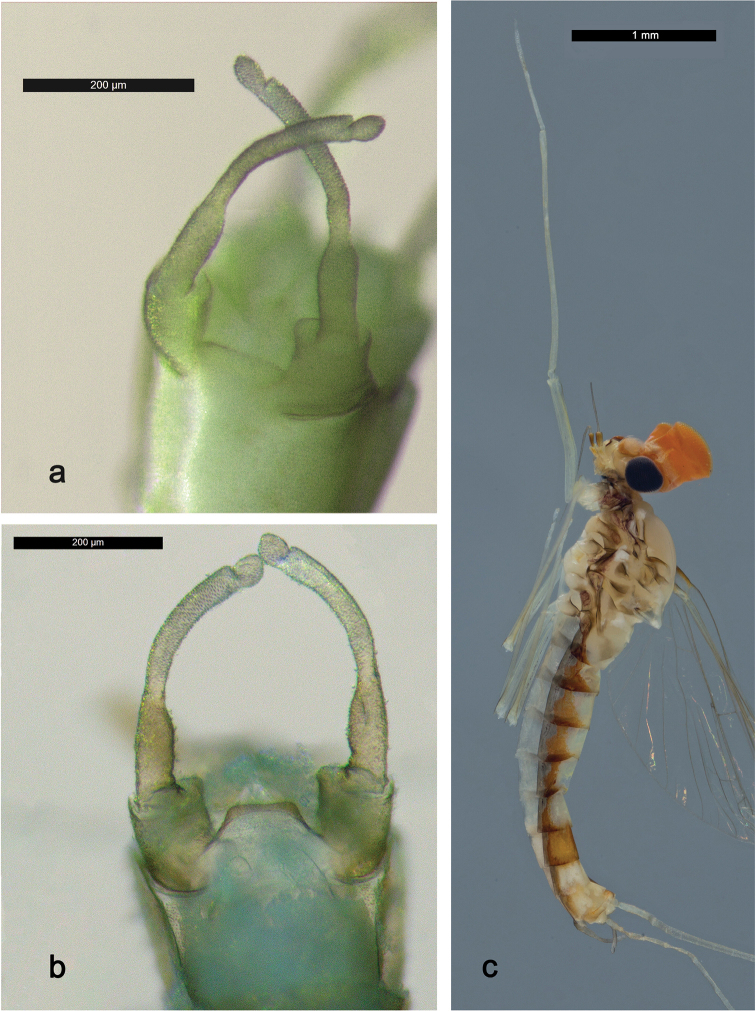
Male imagos: **a***Labiobaetis penan* sp. nov., genitalia **b***Labiobaetis borneoensis*, genitalia **c***Labiobaetis penan* sp. nov., imago, lateral view.

**Table 2.** GPS coordinates of locations of examined specimens.

Species	Locality	GPS coordinates
*L. bakerae* sp. nov.	Brunei	04°32.77'N, 115°09.52'E
		04°32.92'N, 115°09.45'E
		04°33.07'N, 115°09.41'E
*L. penan* sp. nov.	Indonesia: Kalimantan	03°01.67'N, 116°31.08'E
		03°04.07'N, 116°30.43'E
		03°00.17'N, 116°32.40'E
		03°04.93'N, 116°30.97'E
		02°59.37'N, 116°30.77'E
		03°00.15'N, 116°32.42'E
		02°59.20'N, 116°33.18'E
		02°59.48'N, 116°33.48'E
		03°01.80'N, 116°29.80'E
		03°00.95'N, 116°32.27'E
		03°00.95'N, 116°30.52'E
	Brunei	04°32.82'N, 115°09.50'E
		04°32.83'N, 115°09.38'E
		04°32.63'N, 115°08.85'E
	Malaysia: Sabah	06°00.40'N, 116°32.80'E
*L. dayakorum* sp. nov.	Indonesia: Kalimantan	03°00.08'N, 116°30.80'E
		03°00.95'N, 116°32.27'E
		02°59.37'N, 116°30.77'E
*L. borneoensis* (Müller-Liebenau)	Indonesia: Kalimantan	03°04.07'N, 116°30.43'E
		02°59.48'N, 116°33.48'E
		02°59.37'N, 116°30.77'E
		03°00.17'N, 116°32.40'E
		03°01.80'N, 116°29.80'E
		03°04.93'N, 116°30.97'E
		03°00.95'N, 116°32.27'E
		03°01.67'N, 116°31.08'E
		03°00.08'N, 116°30.80'E
		02°59.82'N, 116°31.37'E
	Brunei	04°32.82'N, 115°09.50'E
		04°33.83'N, 115°08.92'E
	Malaysia: Sabah	04°24.08'N, 117°53.35'E
*L. moriharai* (Müller-Liebenau)	Malaysia: Selangor	03°13.07'N, 101°42.75'E
	Malaysia: Sabah	04°24.23'N, 117°53.58'E
	Brunei	04°32.82'N, 115°09.50'E
		04°32.63'N, 115°08.85'E

### Genetics

COI sequences were obtained from two of the three new species (Table 1) as well as from the two other species. In two cases (*L. penan* sp. nov. and *L. borneoensis*) a male imago could be associated with larvae: the COI sequences of the two ontogenetic stages were identical. The genetic distances (K2P) between the species in Borneo are between 19% and 25%, and therefore much higher than 3.5%, which is generally considered as a likely maximal value for intraspecific divergence (Hebert et al. 2003, Ball et al. 2005, Zhou et al. 2010) (Table 3). Very limited genetic distances (between 0% and 1%) were found between specimens of the same species, as in *L. penan* sp. nov., *L. borneoensis*, and partly in *L. bakerae* sp. nov. The only exception is *L. bakerae* sp. nov.: in this species one larva has a distance of 6% from the two others, despite being collected in the same area and having no morphological difference.

**Table 3.** Genetic distances (COI) between sequenced specimens, using the Kimura 2-parameter.

			1	2	3	4	5	6	7	8
**1**	*L. bakerae* sp. nov.	larva								
**2**	*L. bakerae* sp. nov.	larva	0.06							
**3**	*L. bakerae* sp. nov.	larva	0.06	0.01						
**4**	*L. penan* sp. nov.	larva	0.22	0.20	0.20					
**5**	*L. penan* sp. nov.	larva	0.22	0.20	0.20	0.00				
**6**	*L. penan* sp. nov.	imago	0.22	0.20	0.20	0.00	0.00			
**7**	*L. borneoensis* (Müller-Liebenau)	larva	0.20	0.19	0.19	0.21	0.21	0.21		
**8**	*L. borneoensis* (Müller-Liebenau)	imago	0.20	0.19	0.19	0.21	0.21	0.21	0.00	
**9**	*L. moriharai* (Müller-Liebenau)	larva	0.25	0.22	0.23	0.22	0.22	0.22	0.20	0.20

## Discussion

For the assignment of the new species to *Labiobaetis* we are referring to Kluge and Novikova (2014), Müller-Liebenau (1984a) and McCafferty and Waltz (1995). *Labiobaetis* is characterized by a number of derived characters, some of which are not found in other taxa (Kluge and Novikova 2014): antennal scape sometimes with a distolateral process (Fig. 6g); maxillary palp two segmented with excavation at inner distolateral margin of segment II, excavation may be poorly developed or absent (Kaltenbach and Gattolliat 2019: figs 1o–q); labium with paraglossae widened and glossae diminished; labial palp segment II with distomedial protuberance (Kaltenbach and Gattolliat 2019: fig. 1g–n). All these characters vary and may be secondarily lost (Kluge and Novikova 2014). The concept of *Labiobaetis* is also based on additional characters, summarized and discussed in Kaltenbach and Gattolliat (2018, 2019).

Two of the three new species (*L. bakerae* sp. nov., *L. penan* sp. nov.) belong to the rather large *sumigarensis* group and the third one (*L. dayakorum* sp. nov.) to the *operosus* group (Müller-Liebenau and Hubbard 1985, Kaltenbach and Gattolliat 2019). *Labiobaetis bakerae* sp. nov. and *L. penan* sp. nov. can be distinguished by the number of clavate setae forming an arc on the dorsal surface of the labrum (13–15 in *L. bakerae* sp. nov., ca. 22 in *L. penan* sp. nov.), the number of setae at the dorsal margin of the femur (8–11 in *L. bakerae* sp. nov., 15–19 in *L. penan* sp. nov.) and the presence of split tips of the marginal spines of the paraproct in *L. penan* sp. nov. (Fig. 4h). *Labiobaetis bakerae* sp. nov. is morphologically closely related to *L. jacobusi* Kubendran and Balasubramanian from India and *L. geminatus* (Müller-Liebenau and Hubbard) from Sri Lanka (Müller-Liebenau and Hubbard 1985, Kubendran et al. 2015). From the first species *L. bakerae* sp. nov. is different in the shape of the labial palp, the longer maxillary palp (compared to galea-lacinia) and the shorter medial tuft of the hypopharynx (Fig. 1f–h; Kubendran et al. 2015: figs 44, 47, 48). From the second species, *L. bakerae* sp. nov. differs by the very poorly developed distolateral scape process (rather well developed in *L. geminatus*), the shape of the labial palp (distomedial protuberance of segment II more slender and with a clearly concave distal outer margin in *L. geminatus*), the distinct denticles between prostheca and mola of the left mandible (hardly visible in *L. geminatus*), the maxillary palp with a pronounced distolateral excavation (less developed in *L. geminatus*) and the shape of the triangular spines at anterior margin of tergum IV (generally much wider than long; as wide as long in *L. geminatus*, with pronounced points) (Figs 1b, g, h, 2d, g; Müller-Liebenau and Hubbard 1985: figs 5b, d, e, g, 22). The third new species, *L. dayakorum* sp. nov., is morphologically close to *L. paraoperosus* Kaltenbach and Gattolliat from Sumatra, but differentiated in the following characters: thorax and abdomen of *L. dayakorum* sp. nov. dorsally uniform brown (Fig. 11a) and with a distinct pattern in *L. paraoperosus* (Fig. 11b), shape of the labial palp (Figs 5h, 7d), denticles of the right mandible (4+1+3 in *L. dayakorum* sp. nov., 4+3 in *L. paraoperosus*), and size and shape of the hindwing pads (Figs 6h, 7e).

**Figure 14.**
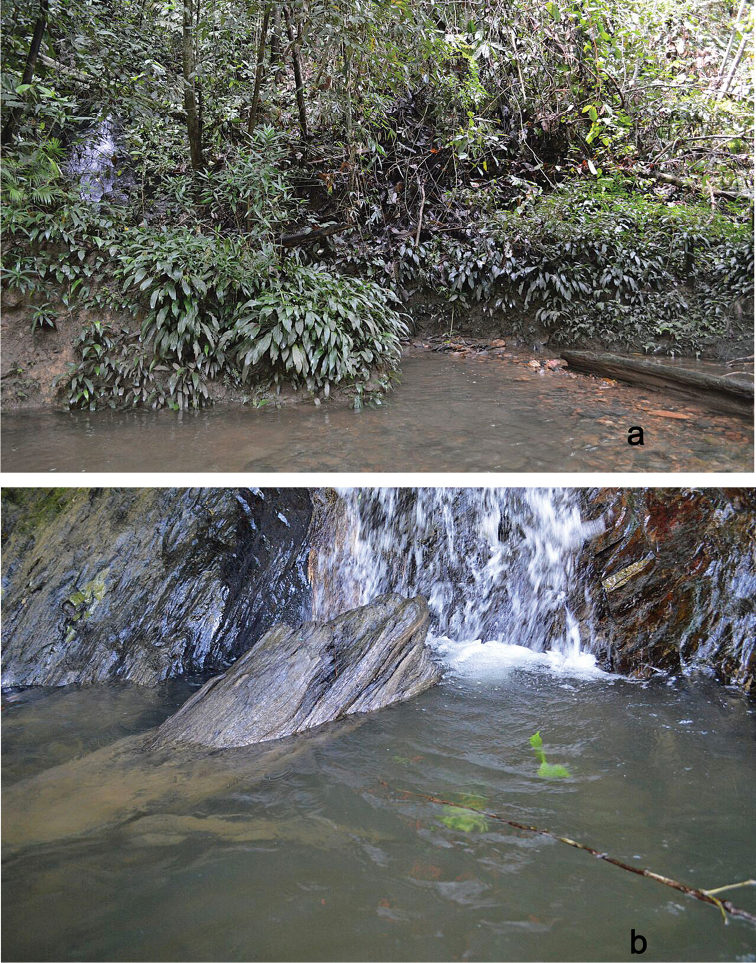
Larval habitats: **a, b***Labiobaetis bakerae* sp. nov., photos Kate Baker.

**Figure 15.**
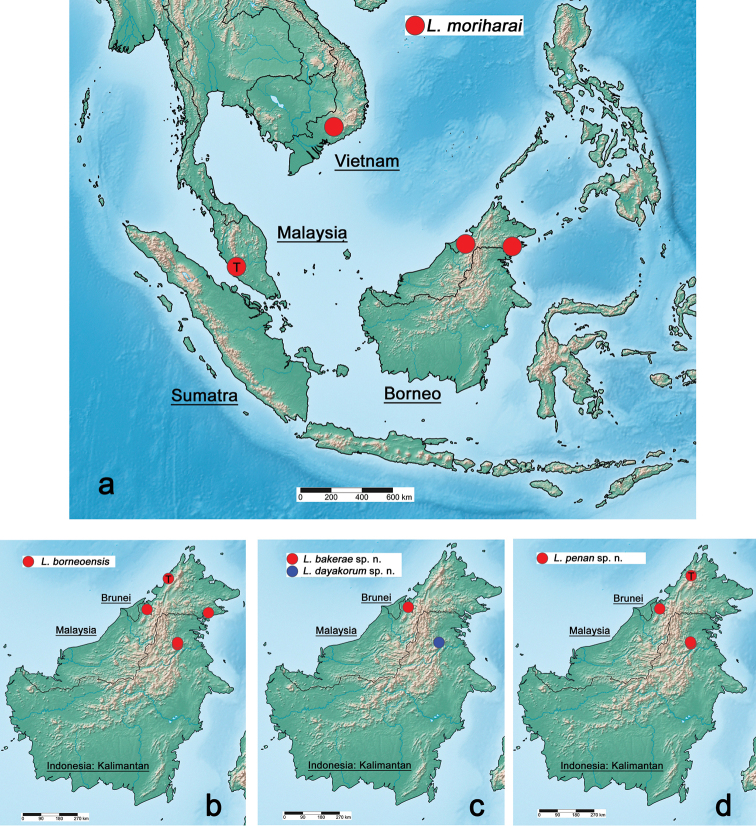
Distribution of *Labiobaetis* in Borneo: **a***Labiobaetis moriharai***b***Labiobaetis borneoensis***c***Labiobaetis bakerae* sp. nov. and *Labiobaetis dayakorum* sp. nov. **d***Labiobaetis penan* sp. nov. T: type locality.

In general, the genetic distances between the different species of *Labiobaetis* are rather high in Borneo, between 19% and 25% (K2P, Table 3), which is in line with the genetic distances found in New Guinea (avg. 22%; Kaltenbach and Gattolliat 2018) and Indonesia (11%-24%; Kaltenbach and Gattolliat 2019). Ball et al. (2005) reported a mean interspecific, congeneric distance of 18% for mayflies from the United States and Canada.

The intraspecific distances are mostly very low as expected, ranging from 0% to 1% (K2P). This result is certainly biased as it is based on a limited number of sequenced specimens per species, which were mostly from a single population. But there is one exception, *L. bakerae* sp. nov., where one specimen has an intraspecific distance of 6% to another specimen of the same population as well as to a specimen of another population. Compared to the usual distances between different *Labiobaetis* species in that region and because there is no morphological difference, this distance is surprising, but can be still considered as intraspecific. Ball et al. (2005) also reported a case with 6% intraspecific distance in a mayfly in North America and intraspecific K2P distances of more than 3.5% are also not uncommon within Plecoptera (Gill et al. 2015, Gattolliat et al. 2016).

In addition to the five species cited in this paper, we obtained two additional COI sequences with clearly interspecific genetic distance to other specimens with similar morphology. In one case, one specimen is highly similar to *L. borneoensis*, but with a K2P distance of 16%. In the other case, one specimen is morphologically very close to *L. penan* sp. nov. and partly damaged, but with a K2P distance of 22%. Because of the limited amount of material and the absence of morphological support, they have to remain species hypotheses for now without further treatment in this paper. Additional material will be necessary to confirm their status in the future. We also have specimens of two additional undescribed species, which have some morphological differences to their closest species. Unfortunately, the material is insufficient or partly damaged and we could not extract DNA. We therefore also refrain to describe them.

The number of sampled localities and different habitats is still very limited and there are large regions, especially in mountainous areas, without any collection activities so far (Fig. 15). In addition, we have four species hypotheses based on genetics only or based on morphological differences without genetics, which may be confirmed as valid species in the future. Therefore, we may assume that the number of *Labiobaetis* species in Borneo will continue to increase substantially with further collections in the future. Thereby, inter-disciplinary collaborations between ecologists and taxonomists may contribute to the discovery of new species in these remote, tropical regions (Baker et al. 2019).

## Supplementary Material

XML Treatment for Labiobaetis bakerae

XML Treatment for Labiobaetis penan

XML Treatment for Labiobaetis dayakorum

XML Treatment for Labiobaetis borneoensis

XML Treatment for Labiobaetis moriharai
